# Recent progress in low‐swellable polymer‐based smart photonic crystal sensors

**DOI:** 10.1002/smo.20230018

**Published:** 2023-12-07

**Authors:** Yong Qi, Shufen Zhang

**Affiliations:** ^1^ State Key Laboratory of Fine Chemicals Frontier Science Center for Smart Materials Dalian University of Technology Dalian China

**Keywords:** low‐swellable polymer, photonic crystal sensors, response, structural color, structural design

## Abstract

Low‐swelling polymers (LSPs) generally refer to materials with a low solvent absorption ratio or volume expansion rate at swelling equilibrium. LSPs with exceptional responsiveness could be upgraded to smart sensors with structural color self‐reporting by bridging photonic crystals (PCs). Based on the regulation of swelling to effective refractive index, lattice spacing, the order‐disorder arrangement of nanostructures, and incident/detection angle, the structural color feedback of smart photonic crystal sensors (SPCSs) can quantitatively and visually reveal the stimulus, which greatly promotes the interdisciplinary development of nanophotonic technology in the fields of chemical engineering, materials science, engineering mechanics, biomedicine, environmental engineering, etc. Herein, to clarify the role of the photonic structures and polymer molecules in high‐performance SPCSs, LSP‐based SPCSs are summarized and discussed, including general swelling mechanisms, color change strategies, structural design, and typical functional applications. It aims to figure out the combination rule between PC structures and LSPs, optimize the design of PC structures, and expound the corresponding structural color sensing mechanisms, inspiring the fabrication of next‐generation SPCSs. Finally, perspectives on future structural design and sensing applications are also presented. It is believed that SPCSs are multifunctional nanophotonic tools for the interdisciplinary development of numerous engineering fields in the future.

## Graphical abstract

The recent progress on LSP‐based SPCSs was summarized, including general swelling mechanisms, color change strategies, structural design, and typical sensing applications. It aims to clarify the role of photonic structures and polymer molecules in SPCSs, figure out the combination rule between PCs and LSPs, and expound corresponding structural color sensing mechanisms, inspiring the fabrication of next‐generation SPCSs.

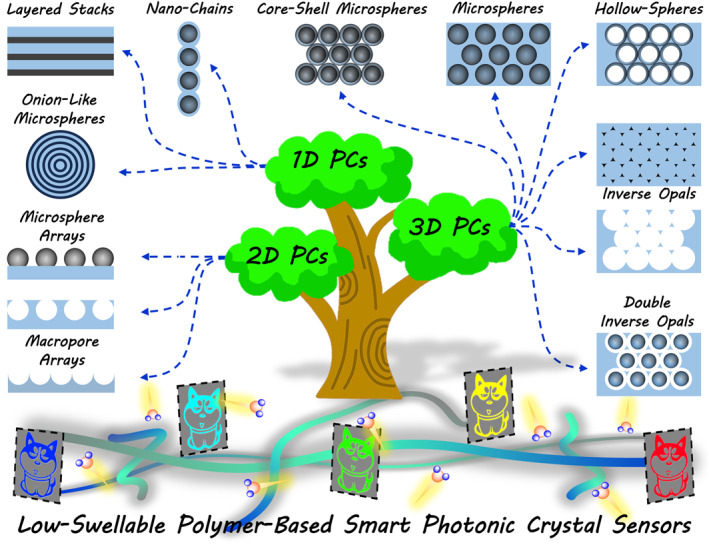

## 1 INTRODUCTION

Gorgeous colors can always make people revel in the fairy tale world, and these colors usually include chemical colors and structural colors. The former results from the absorption or emission of light by molecules, while the latter relies on interference, scattering, or diffraction between light and micro‐nano structures.[^1^, ^2^, ^3^] Among them, structural color has attracted much attention due to its stability and durability against photobleaching or photochemical degradation resulting from its robust structure. The micro‐nano structures in nature provide design inspiration for researchers all the time.[^4^, ^5^, ^6^] A series of bioinspired artificial structural color materials are emerging in the fields of biomedicine,^[7]^ chemical engineering,^[8]^ materials science,^[9]^ mechanics, optics,^[10]^ and so on. Photonic crystals (PCs), as the most important structural color materials, are micro‐nano structures formed by periodic arrangement of media of different refractive indices, displaying photonic band gaps (PBG, reflecting light of specific wavelengths).[^11^, ^12^, ^13^] PCs show bright vivid colors, so‐called structural colors, when PBG appears in the visible light range. Such materials can generally be divided into one‐dimensional (1D, layered stack nanostructures in one direction),^[14]^ two‐dimensional (2D, possessing periodic structure array in two directions),^[15]^ and three‐dimensional (3D, nanostructures ordered periodically in three spatial directions) PCs.^[16]^ PCs, as smart materials with built‐in structural color sensing, have been attracting much attention.[^17^, ^18^, ^19^, ^20^, ^21^]

Most current stimulus‐responsive PCs are structured polymers fabricated by template replication or negative replication, especially Low‐swelling polymers (LSPs).[^22^, ^23^] Based on the limited visible range, usually a small degree of swelling can cause the diffraction displacement of PC to cover the entire visible region.^[24]^ Therefore, high‐swellable polymers (HSPs, generally refer to materials with a high solvent absorption ratio or volume expansion rate at swelling equilibrium) and anti‐swelling polymers (ASPs, generally refer to materials with almost no solvent absorption or volume expansion at swelling equilibrium) are generally less considered. HSPs usually take a long time to reach swelling equilibrium, and the reflection wavelength corresponding to the equilibrium state is most likely in the infrared region. The instability of some intermediate states of the corresponding PCs increases the uncertainty of sensing. Due to the almost constant volume of the ASPs‐based PCs, it is very difficult to show a wide range of reflection wavelength changes, showing limited structural color information. LSP‐based smart photonic crystal sensors (SPCSs) integrate the structural advantages of PCs and the responsive characters of the polymer, showing amazing properties of the visual backtracking stimulus and playing irreplaceable roles in many interdisciplinary disciplines.^[25]^ In response to external stimuli, the physical or chemical properties of the LSP undergo dynamic changes, resulting in reversible changes in micro‐nano structures and triggering changes in structural color. PBG of SPCSs is quite sensitive to the changes in some chemical or physical quantities in the environment and can feedback on the type, activity, concentration, and other parameters of analytes through the change of effective refractive index,^[26]^ lattice spacing,^[27]^ the order of nanostructures,^[28]^ and incident/detection angles.^[29]^ This makes SPCSs particularly useful in a wide range of discipline fields involving sensing.[^30^, ^31^, ^32^]

**FIGURE 1:**
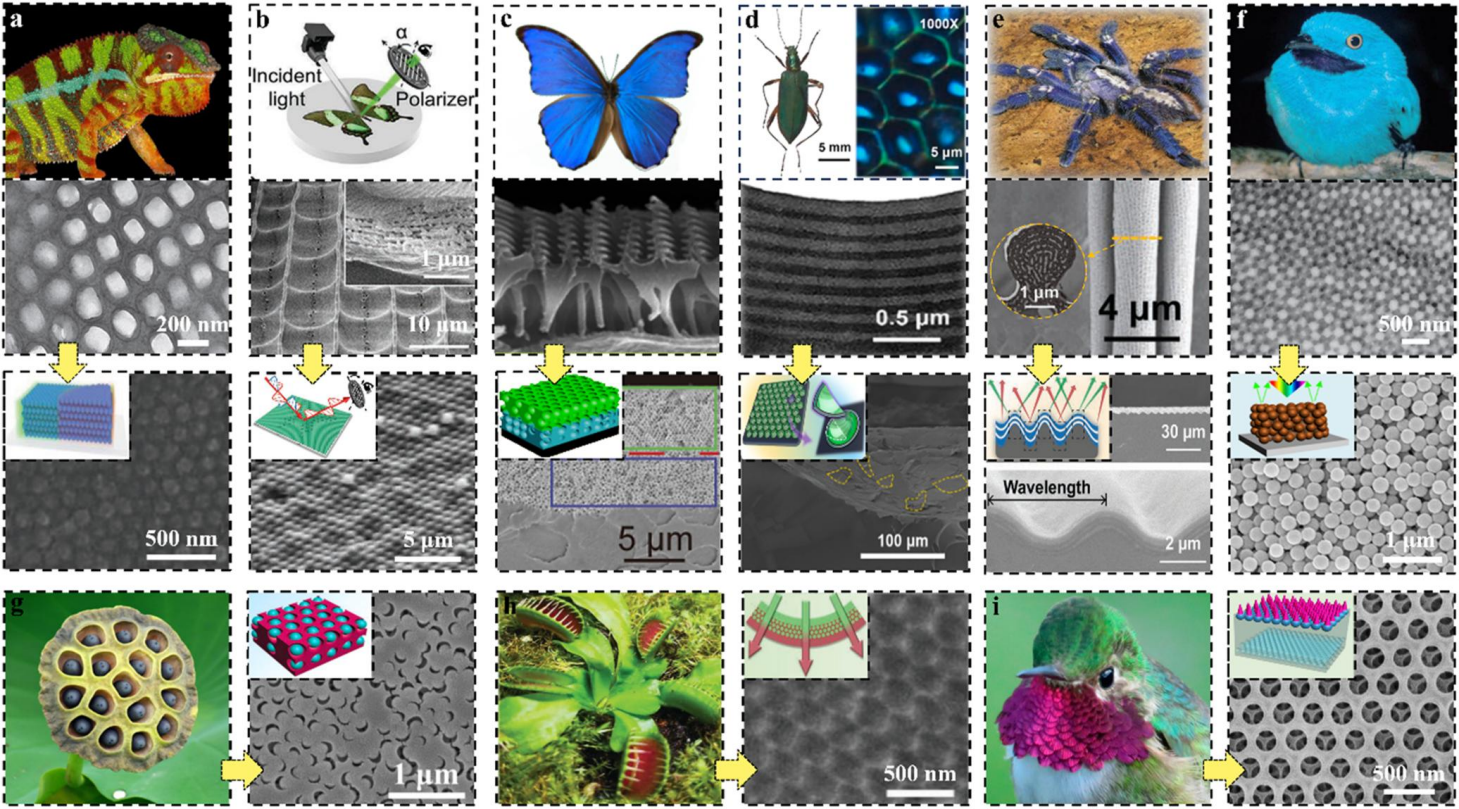
Structure in nature and corresponding bionic artificial structure color materials. (a) Neatly arranged guanine crystals in chameleon skin^[35]^ (Copyright 2015, Springer Nature) and corresponding artificial SiO_2_‐coated ZnS PCs,^[36]^ Copyright 2023, Wiley‐VCH; (b) The hierarchical micro‐nanostructures structure of *Papilio palinurus* butterfly wings and artificial nanoimprinted 2D photonic architectures,^[37]^ Copyright 2022, Wiley‐VCH; (c) The hierarchical ordered and quasiamorphous arrays of *Morpho didius* butterfly wings and the artificial bilayer inverse heterostructures,^[38]^ Copyright 2018, American Chemical Society; (d) The hexagonal pits arrays with multilayered structures in the male beetle *Chlorophila obscuripennis* and bioinspired 1D photonic bowl‐arrays,^[39]^ Copyright 2022, Elsevier; (e) The asymmetric wrinkling protrusions in the hair of *Poecilotheria metallica* spider and artificial wrinkled photonic elastomer,^[40]^ Copyright 2022, Wiley‐VCH; (f) The spherical nanostructures in the plumage of the plum‐throated *Cotinga maynana* ^[41]^ (Copyright 2009, Royal Society of Chemistry) and artificial amorphous photonic structures,^[42]^ Copyright 2019, American Chemical Society; (g) Lotus seedpod and artificial double inverse opal structures,^[43]^ Copyright 2021, Elsevier; (h) Venus flytrap and artificial bilayer structural color hydrogel,^[44]^ Copyright 2019, Wiley‐VCH; (i) Broad‐tailed hummingbird and artificial Janus photonic soft actuator,^[45]^ Copyright 2022, Royal Society of Chemistry.

PC structures are the foundation of preparing SPCSs. Most of the related artificial micro‐nano structures are inspired by nature.[^33^, ^34^] Biological evolution has produced a wide variety of natural photonic structures that produce specialized optical signals to be used as warning signals, aid in camouflage, or allow thermoregulation. The interesting light manipulation mechanisms have inspired people to imitate and fabricate artificial photonic structures. For example, the neatly arranged guanine crystals in chameleon skin^[35]^ inspired the design of polymer‐based structural color materials embedded with opal PCs^[36]^ (Figure 1a); the light polarization effect of artificial arrayed 2D PC derived from the concave micro‐nano structure of *Papilio palinurus* butterfly wings^[37]^ (Figure 1b); the hierarchical ordered and quasiamorphous arrays of *Morpho didius* butterfly wings^[38]^ inspired the construction of bilayer inverse opal (Figure 1c); the hexagonal pits arrays with multilayered structures in the male beetle *Chlorophila obscuripennis* ^[39]^ inspired the preparation of bowl‐shaped 1D PC (Figure 1d); the asymmetric wrinkling protrusions in the hair of *Poecilotheria metallica* spider^[40]^ inspired the design of 1D PC with wrinkled surface (Figure 1e); the spherical nanostructures in the plumage of the plum‐throated *Cotinga maynana* ^[41]^ inspired spraying microspheres to prepare angle‐independent structural color materials^[42]^ (Figure 1f); lotus seedpod inspired preparation of inverse opal embedded with microspheres^[43]^ (Figure 1g); artificial bilayer structurally colored hydrogel actuator inspired by Venus flytrap^[44]^ (Figure 1h); broad‐tailed hummingbird feather‐inspired inverse opal with angle‐dependent retroreflection^[45]^ (Figure 1i), etc. Based on these structural foundations, various SPCSs could be obtained by incorporating LSPs.

Over the past few decades, different kinds of LSP‐based SPCSs, including temperature,^[46]^ humidity,^[47]^ pH,^[48]^ ions,^[49]^ molecules,^[50]^ vapors,^[51]^ etc., have been developed. The advances in LSP‐based SPCSs could be summarized in several areas. The first is template self‐assembly. Corresponding colloidal PC templates are generally fabricated by the methods of vertical deposition,^[52]^ evaporation self‐assembly,^[53]^ shear‐induced self‐assembly,^[54]^ interface self‐assembly,^[55]^ solvent wrapped metastable self‐assembly,^[56]^ magnetic field induced self‐assembly,^[57]^ electric field induced self‐assembly,^[58]^ dip coating,^[59]^ bar‐coating,^[60]^ spin‐coating,^[61]^ drop‐casting,^[62]^ spray coating,^[63]^ Mayer rod coating,^[64]^ resist‐screen printing,^[65]^ inkjet printing,^[66]^ 3D printing,^[67]^ etc. Several pieces of representative literature are strongly recommended to understand the self‐assembly of PC templates.[^68^, ^69^, ^70^, ^71^, ^72^, ^73^, ^74^, ^75^] The second is the selection of polymer materials. Both the hydrophilicity and flexibility of the polymer could be adjusted through reasonable design.^[76]^ The swelling properties of the polymer are also included. According to the detection requirements, special responsive units can also be introduced, such as recognition sites,^[77]^ fluorophores,^[78]^ ionic liquids,^[79]^ reversible covalent bonds,^[80]^ coordination bonds,^[81]^ etc. The third is the surface structure design. Carefully designed microtopography can often change the final physicochemical properties and optical effects of SPCSs,[^82^, ^83^, ^84^] thus improving user experience or reducing detection errors. The fourth is the screening of micro‐nano structures for fabricating PC sensors. Depending on the usage scenario and the desired effect, different kinds of PC structures are selected to maximize the advantages of the combination between PC and polymers.

In addition to traditional detection, LSP‐based SPCSs can also be used in smart displays,^[57]^ wearable devices,^[85]^ soft actuators,^[86]^ drug release/delivery,^[87]^ and organs‐on‐chips.^[88]^ Through programmatic design, SPCSs create beautiful patterns in response to different stimuli to inspire the construction of smart displays or anti‐counterfeiting materials. The combination of LSP‐based SPCSs and flexible substrates can give wearable devices structural color self‐reporting performance, especially real‐time sensing of human parameters.[^89^, ^90^, ^91^] The combination of LSP‐based SPCSs and soft actuators provides a new mechanism for actuating methods on the one hand and optical reading of mechanical parameters on the other hand.[^92^, ^93^, ^94^] For applications of drug delivery and organ‐on‐chips, LSP‐based SPCSs can not only report the degree of drug release in real time but also dynamically monitor the drug activity on biological tissues.[^95^, ^96^, ^97^]

Although the swelling properties of polymers are widely used in SPCSs, the mechanism of low‐swelling‐triggered structural color change, the rational design of micro/nanostructures, interdisciplinary theoretical exploration of sensing, and functional applications still need to be summarized systematically. Herein, the recent progress in LSP‐based SPCSs was summarized and discussed. Firstly, the general color change strategy of responsive PCs is introduced, which is the theoretical basis for the regulation of color change of LSP‐based SPCSs. Secondly, the typical sensing strategies of PCs are summarized, including patterning, fluorescence enhancement, dynamic reflection spectra, and polymer swelling. Thirdly, the swelling mechanism of polymers is recommended from the molecular level. Several popular swelling types are discussed in detail. Fourthly, according to the dimension and structure type of PCs, several popular SPCSs are discussed in detail, focusing on the structural design and the sensing applications. Finally, the present situation, challenges, and prospects of LSP‐based SPCSs are discussed objectively. It is believed that multifunctional LSP‐based SPCSs are powerful nanophotonic technology to transform imperceptible microscopic information into macro color signals for the interdisciplinary development of numerous disciplines in the future.

## 2 COMMON STRATEGIES FOR THE COLOR SENSING OF SPCSs

Such SPCSs contain 1D, 2D, and 3D PCs. The change of structural color is mainly due to the change of effective refractive index (*n*_eff_) and micro‐nano structures of PCs.^[98]^ Among them, 1D PCs are layered structures composed of high (*n*_h_) and low (*n*_l_) refractive index media (polymer or inorganic particles) alternately stacked and assembled in 1D space. The obtained maximum diffraction wavelength (*λ*_max_) and peak intensity (*R*) of 1D PCs are related to the thicknesses of the two media (*d*_h_ and *d*_l_ represent the thicknesses of high and low refractive index media, respectively), satisfying the 1D Bragg equation,^[99]^

(1) $$m\lambda_{\max}=2\left(n_{l}d_{l}\;\cos\;\theta_{l}+n_{h}d_{h}\;\cos\;\theta_{h}\right)$$

(2) $$R={\left(\frac{1-Y}{1+Y}\right)}^{2}\times 100\%,Y={\left(\frac{n_{h}}{n_{l}}\right)}^{N-1}\frac{n_{h}^{2}}{n_{s}}$$

where *θ*_l_, *θ*_h_ are the incidence angles of the light, *m* is an integer, *N* and *n*_s_ represent the periods of the 1D PCs and the refractive index of the substrate, respectively. According to formulas (1) and (2), the *λ*_max_ is regulated by the thickness and refractive index of mediums with high and low refractive index. The *R* is also affected by *n*_h_/*n*_l_ or *N*. If a LSP is introduced, 1D PCs can display different structural colors according to the swelling state by changing the Bragg parameters mentioned above. By establishing the quantitative relationship between swelling and stimulus source, some unknown stimulus conditions could be deduced according to structural color, which is called structural color sensing.

For 2D PCs, it is a kind of micro‐nano array composed of media with different refractive indices arranged periodically in 2D space. The corresponding structural color is derived from the scattering and diffraction of the 2D array^[100]^ and can usually be modulated by the substrate.^[101]^ 2D PCs produce angle‐dependent retroreflection on the same side of the light source. The theoretical diffraction wavelength (*λ*) could be calculated based on the planar grating formula.^[102]^

(3) $$m\lambda =d_{\text{gs}}\;\left[\sin\;\alpha +\sin\left(\beta +x\right)\right]$$

(4) $$m\lambda =\sqrt{3}\;D\;\sin\;\alpha$$

where *d*_gs_ is the spacing of groove (for 2D microsphere arrays, represents the half period of the trigonal lattice, *d*_gs_ *=* $\frac{\sqrt{3}}{2}$ *D*, and *D* is the microsphere diameter or 2D particle spacing). *α*, *β*, and *x* represent the corresponding incident angle, diffraction angle, and the free constant associated with the discrepancy in diffraction energy, respectively. Most commonly, the *λ* at the position of incident light could be approximated by formula (4).^[100]^ Moreover, *D* could be calculated according to the diameter of the Debye diffraction ring (*D*_debye_) of such 2D PCs.^[103]^

(5) $$D=\frac{2\lambda_{\text{laser}}\sqrt{4h^{2}+D_{\text{debye}}^{2}}}{\sqrt{3}D_{\text{debye}}}$$

where *λ*_laser_ is the wavelength of the laser, and *h* is the vertical distance from 2D PC to the screen. According to formulas (3) and (4), the *λ* could be controlled by *D*, *α*, and *β*. Due to the angle dependence of 2D PCs, the change of *D*_debye_ is more suitable for quantitative analysis of the change of stimulus factors. Because the spacing of monolayer arrays is more easily modulated by the substrate, polymer‐based 2D PCs are particularly popular in the sensing analysis of swelling stimulation.

For 3D PCs, it is a kind of micro‐nano structure composed of media with different refractive indices arranged periodically in 3D space. The corresponding structural color is derived from the diffraction of the 3D structures. The *λ*_max_ generally conforms to Bragg's law,^[104]^

(6) $$m\lambda_{\max}=2n_{\text{eff}}d\;\sin\;\theta$$

(7) $$\lambda_{\max}=2n_{\text{eff}}\left(d/m\right)\cos\;\alpha$$

where *d* $\left(=\sqrt{\frac{2}{3}}\;D\right)$ is the lattice spacing, *θ* is the glancing angle between the 3D PC plane and incident light, and *D* is the spacing of microspheres or macropores. The transpositional Bragg equations could also be used (for the first order, *m* = 1),^[105]^

(8) $$\lambda_{\max}=2d{\left(n_{\text{eff}}^{2}-\sin^{2}\;\alpha \right)}^{1/2}$$

(9) $$\lambda_{\max}=1.633D{\left(n_{\text{eff}}^{2}-\sin^{2}\;\alpha \right)}^{1/2}$$

(10) $$n_{\text{eff}}^{2}=\sum n_{i}^{2}V_{i}$$

(11) $$\lambda_{\max}=2d\left(1-v\;\varepsilon_{x}\right)n_{\text{eff}}$$

In artificially prepared PCs, colloidal microspheres or macropores are embedded in the polymer substrate, and the corresponding *n*_eff_ could be approximated as Equation (10).^[106]^ *n*_i_ and *V*_i_ represent the refractive index and corresponding volume fraction of each component, respectively. For elastic PCs, where the PC structure moves with polymer tension and the lattice spacing is modulated by the strain‐stress, *λ*_max_ perpendicular to the tensile direction could be calculated from Poisson's ratio (*v*) and the tensile strain in the *x*‐direction (*ε*_x_) at typical tensile conditions.^[80]^

**FIGURE 2:**
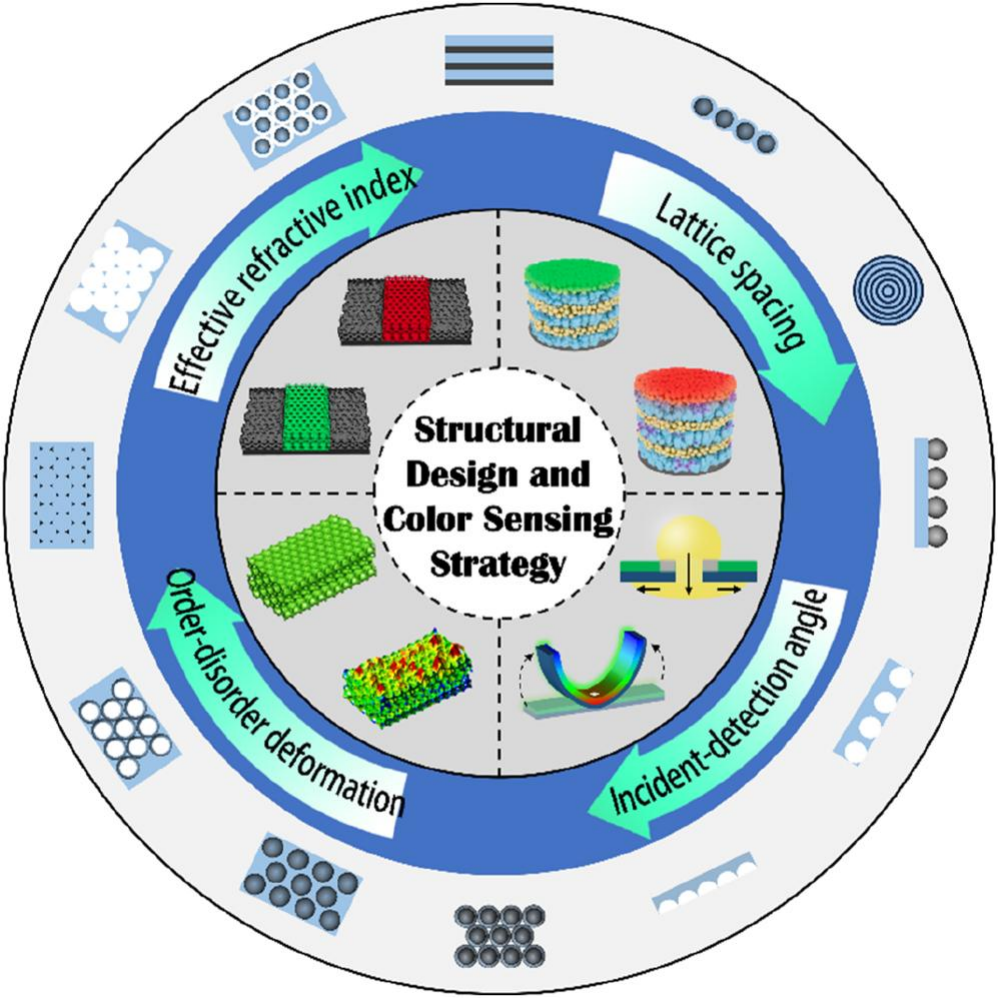
LSP‐based SPCSs, structural design, and color sensing strategy. Lattice spacing,^[61]^ Copyright 2019, Elsevier; Incident‐detection angle,^[45]^ Copyright 2022, Royal Society of Chemistry; Order‐disorder deformation,^[108]^ Copyright 2015, Springer Nature; Effective refractive index,^[109]^ Copyright 2023, Wiley‐VCH.

During a typical stimulus‐response, any change in the Bragg parameters will result in a change in the reflection wavelength.^[107]^ The LSP here exerts a selective or specific response. According to above Bragg's laws, combining the swelling mechanism of the polymer and the set chemical or physical relationship, the corresponding mechanism of structural color change could be roughly deduced. The existing mechanisms of coloration and color change include the change of *d*, incident/detection angle, *n*_eff_, and order‐disorder transition of periodic nanostructures (Figure 2). The change of *d* is determined by the spatial arrangement of the micro‐nano structure of PCs, such as the change of particle spacing during the strain process.^[110]^ The change of incident/detection angle is generally caused by human manipulation or stimulation‐induced system deformation.^[38]^ Changes in *n*_eff_ are usually accompanied by phase transitions or the introduction of new substances, such as penetration or absorption of solvents^[111]^ and gas molecules.^[112]^ The order‐disorder transition of periodic nanostructures generally involves the shape memory properties of polymers.^[113]^

## 3 UNIVERSAL SENSING STRATEGY FOR RESPONSIVE PCs

**FIGURE 3:**
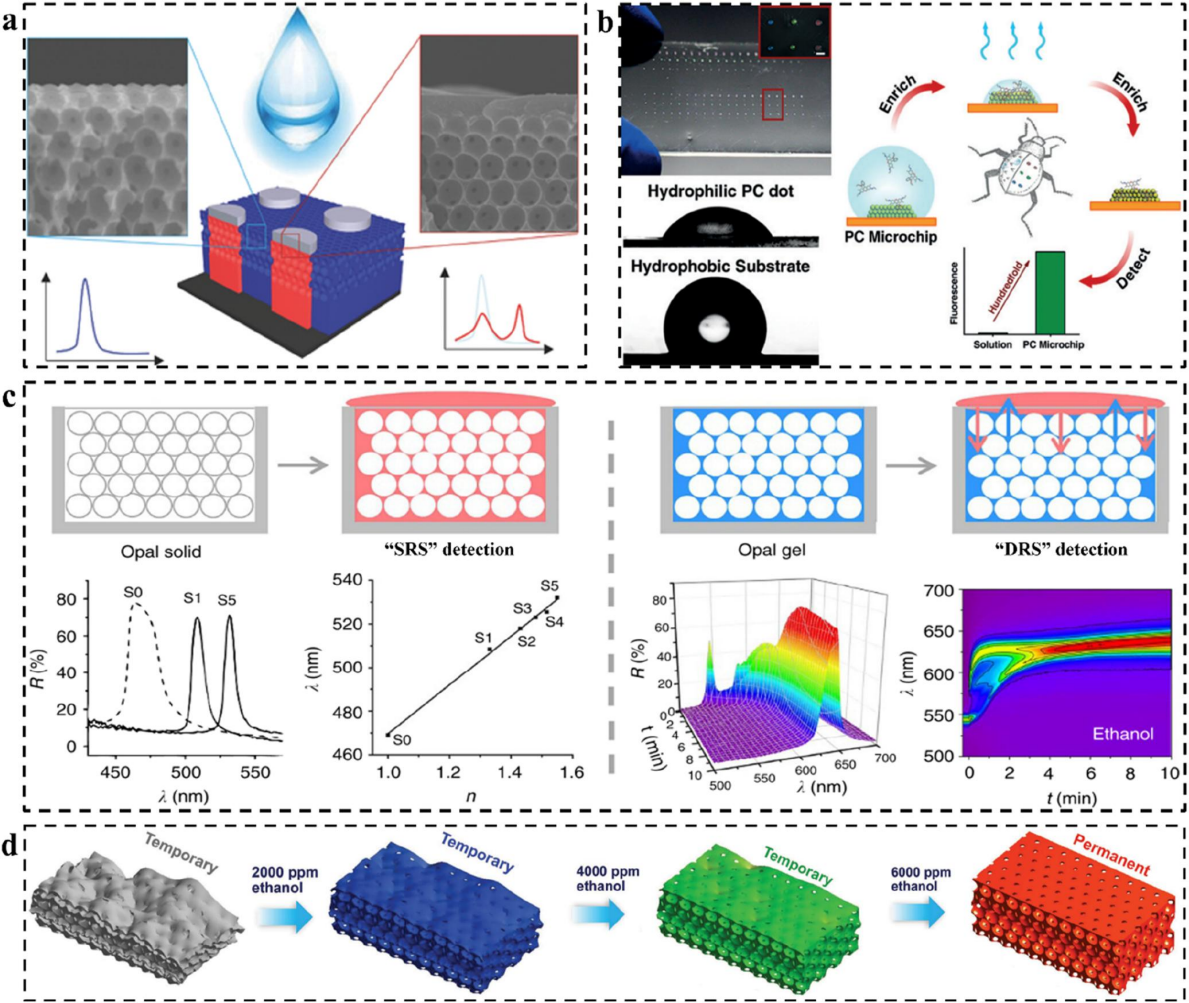
Typical sensing strategies for PCs. (a) Patterned inverse opal PC sensors with different wettability for solvent detection,^[121]^ Copyright 2016, Wiley‐VCH; (b) Fluorescence enhancement on a hydrophilic‐hydrophobic PC microchip,^[115]^ Copyright 2014, Wiley‐VCH; (c) The SRS and DRS analysis for distinguishing solvents,^[122]^ Copyright 2015, Springer Nature; (d) Ethanol detection based on the swelling effect‐induced macropore shape recovery,^[117]^ Copyright 2018, Wiley‐VCH.

The most important feature of the responsive PCs is that the variational color depends on the environmental conditions.^[104]^ The sensing strategy for responsive PCs with polymer and non‐polymer substrates is universal. Color presents in a variety of ways, such as patterning to gradually display,^[114]^ array‐dye combination to enhance the fluorescence signal,^[115]^ dynamic reflection spectra to track the stimulus diffusion in real‐time,^[116]^ and swelling‐induced reversible structural deformation.^[117]^ These methods constitute the general sensing strategy of PCs. For patterning, some important methods should be learned, such as patterned substrate‐induced PC assembly,^[118]^ inkjet printing,^[119]^ selective immobilization and modification,^[120]^ etc. The purpose of these patterning methods is to construct and integrate different response modules to realize the visual detection of different stimuli through programmed coloration or color change.^[25]^ For example, Kim and co‐workers constructed lithographically featured micropatterns on the top surface of hydrophobic inverse opals, which imparts the patterned wettability of such a PC. Only the wetting by a specific solvent can correctly display the encrypted information (Figure 3a).^[121]^ Aizenberg and co‐workers constructed different wetting surfaces by selective hydrophilic patterning of hydrophobic inverse opal PC sensor.^[114]^ Due to the constraint of total free‐energy, liquids with different surface tension (such as ethanol with different concentration) can penetrate different macroporous regions, producing different structural color patterns. Thus, ethanol with different concentrations could be identified by colorimetry. For signal enhancement, PBG of PCs can enhance the fluorescence intensity through reflection,^[123]^ and greatly improve the sensitivity of ultra‐trace detection. For example, Song and co‐workers constructed a hydrophilic PC on a hydrophobic PDMS substrate and enriched the target from the solution to the hydrophilic detection area through evaporation, thus significantly increasing the target concentration in the detection area (Figure 3b).^[115]^

For the analysis of time/spatial resolved reflection spectra, it is necessary to design the PC skeleton and set up the filling system for solvent exchange.^[124]^ The subtle changes of a series of parameters in the system can lead to the change of the reflection spectra according to the diffusion and extraction between the filling liquid and stimulus solvent.^[116]^ Depending on the type of solvent, the time‐dependent dynamic reflection spectrum (DRS) will produce different types of contour maps to identify specific stimulus sources. Based on the geometric features of the DRS maps, Ge and co‐workers achieved the accurate identification of organic solvents, even homologs and isomers (Figure 3c).^[122]^ While traditional static reflection spectrum methods rely only on the final state of the reflection spectrum, DRS analysis can provide detailed variations of spectra, demonstrating deeper sensing potential. This kind of PC sensor can also further increase the span of the maximum reflection wavelength through the swelling effect of the polymer skeleton, which helps to improve the applicability of the DRS map analysis in a sense.

For swelling‐induced reversible structural deformation, those types of PC sensors are usually composed of PC structures and polymers. The swelling is mainly due to the interaction between the polymer and the solvent, and the color change is mainly generated by the PC structures.^[11]^ The swelling of the polymer can result in changes in the *n*_eff_, lattice spacing, structure order, and incident/detection angles of the system. The type of polymer determines the specificity of the SPCSs, for example, polymers with acid‐base groups are usually sensitive to pH,^[125]^ polymers containing photoisomerized groups generally have photo regulatory properties,^[126]^ and polymers containing polyethylene glycol structures are likely to have micro‐nano shape editing properties.^[28]^ By introducing shape memory polymers (SMPs), Jiang and co‐workers realized the detection of ethanol in octane and gasoline through the shape recovery of macroporous skeleton based on the swelling effect of ethanol (Figure 3d).^[117]^ It is obvious that the low‐swelling of the polymer not only does not lead to excessive expansion of the PC lattice but also derives shape memory properties by combining the size effect of the PC. By using the swelling‐controlled shape memory of macropores, the concentration of stimulus was transformed into easily distinguishable color information, which fully reflects the sensing advantage of LSP‐based SPCSs. Polymer‐based SPCSs inherit the responsiveness and structural color sensing of PCs on the one hand and give PCs robust structures to prevent irreversible damage during detection. In addition, polymer‐based SPCSs are easy to carry and store. Since the type of polymer could be set according to the use scenario, LSP‐based SPCSs have received wide attention. Subsequently, this review will introduce and summarize SPCSs in detail from the structural design according to the dimensional categories of PCs.

## 4 TYPICAL SWELLING MECHANISM OF POLYMERS

Typical swelling polymers are generally composed of hydrophilic 3D crosslinked polymer skeleton, which can accommodate a certain amount of solvent.^[127]^ The most common is hydrogels, whose molecular chains generally contain a large number of hydrophilic groups.^[128]^ Dry hydrogels can usually absorb tens of times their dry weight of water or solvent, which is called swelling (an important feature of hydrogels). LSPs are usually prepared under non‐swelling equilibrium conditions, and generally show water absorption and volume increase in response to solvent stimulation, thus changing the original physical or chemical properties of the material.^[129]^ Specifically, stimulus‐response hydrogels can change their swelling ratio in response to changes in external conditions, such as pH and temperature.^[130]^ However, some emerging applications have put forward more stringent requirements for the swelling properties of hydrogels. Such as mechanical and dimensional stability, rather than large expansion. Although hydrogels do not disintegrate in most swelling processes due to their 3D cross‐linked chains,^[131]^ excessive swelling of hydrogels can seriously weaken their mechanical properties. Similarly, in hydrogel‐based PC sensors, excessive swelling may result in a larger lattice spacing, resulting in a maximum reflection wavelength beyond the visible light range, which is almost unnecessary.^[24]^ Therefore, LSPs or ASPs seem to be more concerned in the construction and application of SPCSs.

LSPs, including ASPs, are not clearly defined in the literature. In this review, the swelling mechanism of polymers is summarized for workers in the field of SPCSs in an easy‐to‐understand perspective. More complex swelling theories are beyond the scope of this article. Research on low‐swelling or even non‐swelling hydrogels dates back to the 1960s when Wichterle and Lim developed non‐swelling hydrogels for contact lenses.^[132]^ Later, Lucast et al. clarified the meaning of “low‐swelling”, which opened the prelude to the development of low‐swelling hydrogels.^[129]^ In the early stage, these low‐swelling hydrogels were mainly prepared by increasing the crosslinking density of polymers. High crosslinking density reduced their flexibility and hardened the hydrogels, which inhibited the application of hydrogels. In 2013, Messersmith *et al.* reported hydrogel tissue binders with zero‐ or negative‐swelling.^[133]^ The real breakthrough in low‐ and anti‐swelling hydrogels came in 2014 when Sakai *et al.* reported a non‐swellable polymer without mechanical hysteresis.^[134]^ Since then, a series of multi‐purpose advanced functional hydrogels with high strength,[^135^, ^136^, ^137^] electrical conductivity,[^138^, ^139^] self‐healing,[^140^, ^141^] adhesion,[^142^, ^143^] injectability,^[144]^ and other functions have been actively developed, swelling hydrogels have been rapidly developed.

Low‐swellable could be achieved through H‐bonding, hydrophobic association, ionic complexing, etc. For H‐bonds, especially double and multiple H‐bonds, the binding energy can even be comparable to covalent bonds.^[145]^ It has been calculated that the binding energy of double H‐bonds is much larger than that of singlet H‐bonds (−5.5 and −0.34 kJ/mol, respectively).^[146]^ Double H‐bonds are much more stable than singlet H‐bonds, and hydrogel polymers with double or multiple H‐bonds generally show higher strength.^[147]^ The hydrophobic association of amphiphilic molecules has been proven to play a role as a physical polycrosslinker in the construction of hydrogel polymers with self‐healing and good mechanical properties.^[148]^ Some self‐sealing effects can effectively resist the invasion of solvent molecules and greatly weaken the swelling of the polymer. For ionic complexing, the salt stability of hydrogel polymers made of different positively charged monomers depends on the strength distribution of ionic bonds and the hydrophilic and hydrophobic equilibrium of the network.^[149]^ Low swelling can avoid the excessive swelling of the polymer‐PC composite system and the subsequent irreversible damage to PC structures. LSP‐based SPCSs are rapidly sweeping the field of smart sensing. The detailed swelling theory is shown below.

### 4.1 Evaluation of the polymer swelling

To promote the practical application of LSPs in a wide range of sensing fields, it is necessary to understand the basic swelling mechanism and equilibrium of polymers. Swelling capacity, the so‐called equilibrium swelling degree, is often considered an important “power factor” in polymer hydrogels and is useful in evaluating related swelling behavior.^[129]^ In general, the mass and volume size of the polymer system change during the expansion process, and many characterization parameters are used to measure the swelling capacity of the polymer.[^133^, ^150^] Swelling rate (*ω*, the ratio of solvent absorption mass to initial polymer mass when the polymer reaches swelling equilibrium, formula (12))^[45]^ and equilibrium volume rate (*Q*_v_, *V*_0_, and *V*_e_ are the initial volume of polymer and the volume at swelling equilibrium, respectively, formula (14)))^[151]^ are the most important parameters to measure such swelling. Where *m*_0_ and *m*_e_ are the initial mass of the polymer and the mass at swelling equilibrium, respectively. The *ω* of the polymer at time *t* (the corresponding mass is *m*_t_) can be expressed by formula (13).^[45]^

(12) $$\omega =\frac{m_{e}-m_{0}}{m_{0}}\times 100\%$$

(13) $$\omega =\frac{m_{t}-m_{0}}{m_{0}}\times 100\%$$

(14) $$Q_{v}=\frac{V_{e}}{V_{0}}\times 100\%$$

When the polymer comes into contact with the solvent (such as water), the solvent diffuses into a pre‐existing or dynamically formed space between the polymer chains.^[152]^ The swelling of the polymer produces a larger scale of segmental motion, eventually leading to an increase in the distance between the polymer chains.[^153^, ^154^] The corresponding solvent retention rate (*η*) could be calculated from formula (15). For thin‐film polymers, based on the typical Fickian diffusion model,^[127]^ the swelling mechanism of the copolymer could be determined according to the formula (16). According to the fitting value of swelling exponent (*n*, where *k* represents the swelling constant), the swelling behavior of copolymer could be divided into three theoretical modes: (1) *n* < 0.5, indicating a Fickian type, which could be interpreted as the free diffusion of solvents; (2) 0.5 < *n* < 1, indicating a non‐Fickian type, the solvent diffusion and the motion of copolymer chains jointly regulates the solvents adsorption; (3) *n* > 1, the diffusion system is controlled by the extension of the copolymer chains.

(15) $$\eta =\frac{m_{t}-m_{0}}{{m_{e}-m}_{0}}$$

(16) lnη=lnk+nlnt

### 4.2 Typical driving force of the equilibrium swelling

In the case of water‐responsive polymers, the hydrophilicity of the polymer network creates osmotic pressure and thus results in expansion. The expansion process generally consists of three steps: (1) diffusion of water molecules in the polymer, (2) relaxation of the polymer chain under hydration, and (3) expansion of the polymer network after relaxation.^[155]^ When the polymer swells to a certain extent, it reaches equilibrium with the elastic contraction force (reaction force) generated by the 3D network. At this point, the amount of water absorbed by the polymer reaches its peak (the so‐called equilibrium water content). The network expansion stops and reaches its equilibrium. When there is a change in osmotic pressure or crosslinking density, such as a decrease in crosslinking points due to pH‐induced deprotonation of carboxyl groups in the network or degradation of the network, the balance will be upset. LSP‐based SPCSs make use of this principle. Based on Flory and Rehner's equilibrium swelling theory,[^156^, ^157^] the expansion of a polymer network is a function of the elastic force of the polymer chain and the thermodynamic compatibility between the polymer and water molecules. The free energy (Δ*G*) of a neutral polymer could be expressed as:

(17) $$∆G=∆G_{el}+∆G_{mix}$$

where Δ*G*_el_ and Δ*G*_mix_ represent the contribution of elastic refractive forces and the thermodynamic compatibility of polymer and solvent, respectively. It could be rewritten in terms of chemical potentials. The total chemical potential (*μ*) at the equilibrium conditions should be equal to zero (formula (18)). The change in chemical potential due to elastic forces (*μ*_el_) could be expressed by using the theory of rubber elasticity, while the contribution of mixing to chemical potential (*μ*_mix_) change is determined using the heat and entropy of mixing.^[155]^

(18) $$\mu =\mu_{el}+\mu_{mix}=0$$

**FIGURE 4:**
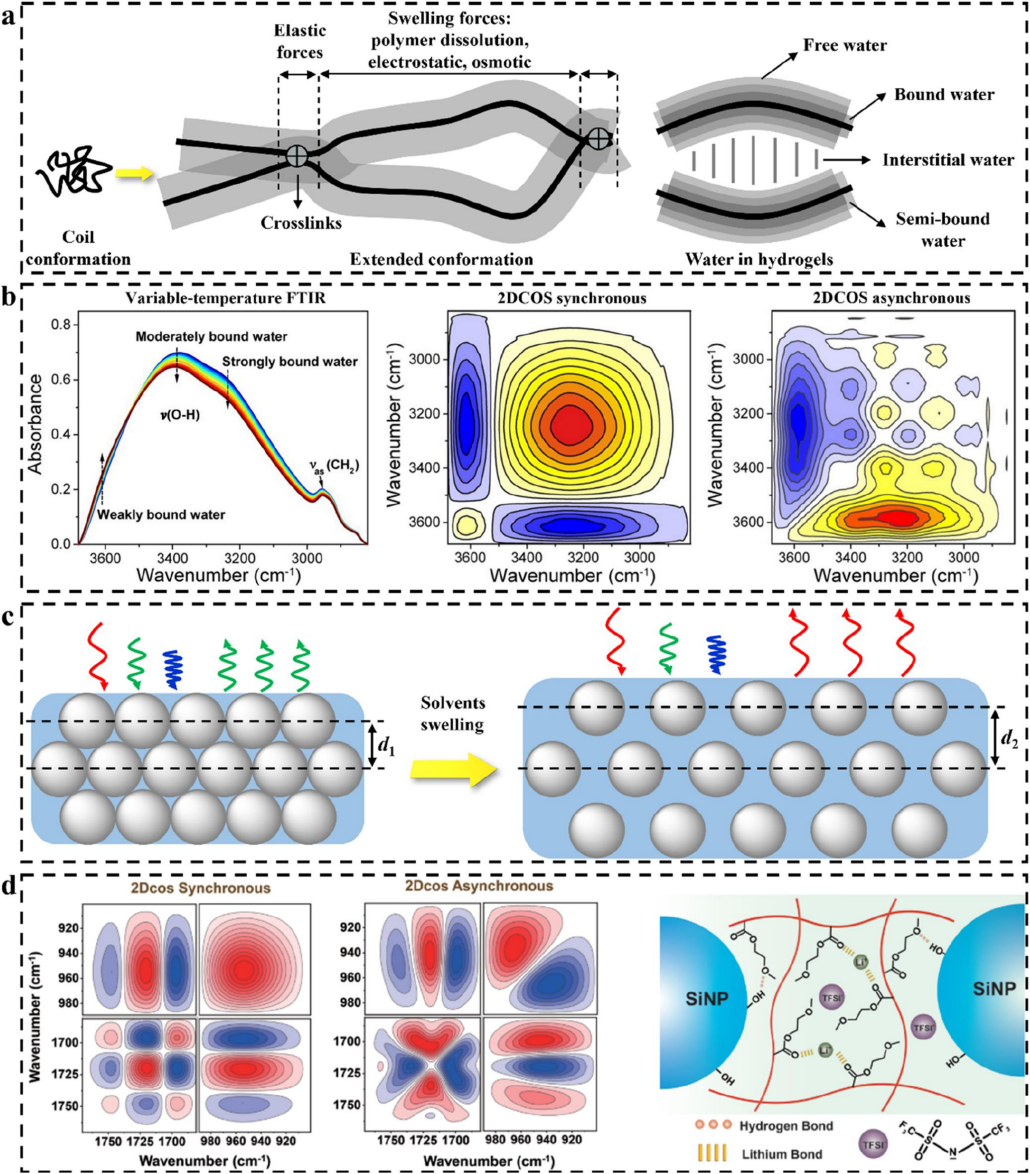
(a) Schematic mechanism of polymer swelling,^[129]^ Copyright 2021, Elsevier; (b) Typical variable‐temperature FTIR spectra and corresponding 2D correlational analysis of polymer during temperature rise,^[158]^ Copyright 2022, Wiley‐VCH; (c) Schematic diagram of solvent‐induced swelling and reflection redshift of a typical SPCS; (d) 2D correlational analysis interprets the internal interactions between the polymer and the microspheres,^[159]^ Copyright 2023, Wiley‐VCH.

As long as the polymer in a non‐equilibrium state is immersed in a solvent such as water, it will spontaneously swell. The driving force of the swelling of the polymer network is mainly from the interaction between water and hydrophilic molecular chains, which weakens the interchain force and promotes the infiltration and diffusion of water into the polymer networks. During the swelling process, water first forms hydrogen bonds (H‐bonds) with polar hydrophilic groups on the chain, producing what is known as primary‐bound water (Figure 4a).^[129]^ The weakly hydrophilic or hydrophobic groups are then exposed and interact with the water molecules to produce secondary‐bound water. These two kinds of bound water together constitute the total bound water. As water permeates, the pores among the polymer chains are filled, and the amount of this water, known as free water, is determined by the porosity of the polymer network.^[160]^ The whole swelling process causes the coiled polymer chain to extend continuously, and the extended conformation increases the distance and volume among the networks.^[161]^ Due to the cross‐linking between the polymer chains, they do not dissolve. At the same time, the 3D spatial extension of the network creates a resistance to expansion, also known as the elastic retractive force. This kind of force resists the reduction of configuration entropy caused by expansion through network shrinkage. The polymer‐water interaction force competes and symbiosis with the elastic retractive force of the network. At the beginning of swelling, the former is greater than the latter, and as the polymer network gradually changes from coiled conformation to extended conformation, the latter gradually increases. When the effects produced by the two cancel each other, the polymer reaches swelling equilibrium.

In addition, the swelling of the polymer is also regulated by the active groups on the chain, which ionize to produce mobile counter‐ions. The difference in the concentration of counter‐ions flowing inside and outside the network promotes solvent osmosis. The ionized groups repel each other due to the same charge, resulting in swelling of the polymer network. At this time, the driving forces of swelling include polymer‐water interaction (osmotic pressure caused by mixing), counter‐ion‐induced osmotic pressure, and interchain electrostatic repulsion.^[129]^ Depending on the response type of polymers, the driving forces for swelling equilibrium are varied.[^162^, ^163^, ^164^] In the electrolyte environment, the strength of the electric field can manipulate the swelling degree of the polymer,^[165]^ and the oxidation‐reduction state can also trigger the wettability change of the polymer, thus changing the swelling state.^[166]^ In the gas atmosphere, the liquid produced by the condensation of the capillary in the polymer channel directly promotes the swelling of the polymer, and the gas pressure is the driving force of the swelling of the whole system.^[112]^ In the acid‐base system, pH can change the sensitive groups (such as carboxyl, amino, etc.) on the polymer chain,^[167]^ thereby regulating the equilibrium potential of polymers, changing the osmotic pressure and swelling state. In organic solution, intermolecular force, H‐bonds, and dipole action can also regulate the adsorption of polymer to solvent and regulate the corresponding swelling.^[139]^ In an ionic liquid condition, the ionic bonds and the associated equilibrium potential act as the driving force of swelling.^[168]^

Depending on the nature of the polymer, swelling is affected by many physicochemical conditions or structural factors,[^129^, ^136^, ^169^, ^170^] including temperature,^[171]^ light,^[172]^ pH,^[173]^ electric field,^[174]^ etc. The polymer containing poly (N‐isopropyl acrylamide) produces different hydrophilic and hydrophobic states at different temperatures, thus adjusting the swelling.^[175]^ The spiropyran‐contained polymer produces phenoxy anions by reversible ring‐opening under light to adjust the equilibrium potential of the system,^[176]^ thus manipulating the swelling. The polymer with micro/nano structures can also use the nano‐size effect to regulate the wettability of pores and directional wetting to achieve anisotropic swelling.^[177]^

In addition, the cross‐linking degree of the polymer is a key factor in determining the swelling ratio. For example, highly cross‐linked hydrogels have smaller mesh sizes and less expansion than loosely cross‐linked hydrogels. The crosslinking density determines the maximum movement of polymer chains. Generally, the higher the cross‐linking density, the more difficult the polymer is to swell (due to the limitation of the interchain binding force, sacrificing the polymer toughness). Specially, as a physical crosslinking method, chain entanglement is also one of the popular regulatory parameters of polymer swelling.^[135]^ Unlike chemical crosslinking, entanglement does not sacrifice the toughness of the material and makes the polymer have excellent mechanical properties.^[137]^ Entanglement restricts the movement of polymer chains during the swelling process by physical crosslinking. The dynamic covalent bonds regulate the cross‐linking density and entanglement by changing the interchain binding force,[^178^, ^179^, ^180^] so as to manipulate the movement of polymer chains during the process of swelling. In short, there are many ways to regulate polymer swelling, and these are the sensing basis of LSP‐based SPCSs.

### 4.3 Noda's rule for analyzing intermolecular action mechanisms

Swelling is a series of physical (and possibly chemical) changes resulting from the interaction between a polymer and a solvent. Characterizations at the molecular level are helpful to elucidate the relevant mechanism of swelling. In the case of water, the interaction mechanism between solvent and polymer chain can be further understood by the real‐time FTIR spectra of wetted polymer samples.[^181^, ^182^, ^183^] Due to the characteristic peaks of some functional groups overlapping each other, it is difficult to interpret spectroscopic intensity fluctuations of copolymers under various external conditions such as pressure, temperature, concentration, time, electric field intensity, etc. As a comprehensive method proposed by Noda,[^184^, ^185^] 2D correlational analysis can intuitively identify overlapping peaks in the original spectra by spreading the spectral bands along a second dimension, which improves the spectral resolution. Wu *et al.* used Noda's rule to determine the order of interaction among functional groups and water in the process of dehydration of polymers, revealing the reasons for some physical properties changes caused by swelling or shrinking of polymers from the molecular level (Figure 4b).^[158]^ 2D correlational analysis can reveal the sequence of subtle changes in molecular or group fragments stimulated by environmental conditions, including the spectral information covered by other characteristic absorptions.[^186^, ^187^] According to the sequence of change events, it can help analyze the corresponding response mechanism of the material. This kind of 2D correlational analysis is particularly useful when revealing the swelling or shrinking of polymers at the molecular level.^[188]^ Noda's rule for the generalized 2D correlational analysis could be summarized as follows.

Generally, 2D synchronous and asynchronous spectra could be obtained based on the dynamic 1D spectra. The 2D synchronous and asynchronous spectra reflect the relative degree of in‐phase and out‐of‐phase responses, respectively. The 2D synchronous spectra are symmetrical concerning the diagonal of the map.^[185]^ Non‐diagonal peaks, so‐called cross peaks, might be positive or negative, representing a simultaneous or coincidental change in the intensity of two measured characteristic peaks. The 2D asynchronous spectra are asymmetrical concerning the diagonal of the map. Asynchronous spectra show only non‐diagonal cross peaks, which can also be positive or negative. A cross peak in an asynchronous map occurs only when there is an out‐of‐phase change (i.e. delay or acceleration) in the intensity of the two dynamic characteristic spectra. Those features are particularly useful in distinguishing overlapped peaks produced by different spectral origins or moieties.

For the characteristic wavenumbers *v*_1_ and *v*_2_ (cross‐peaks, assuming *v*_1_ > *v*_2_), if the correlation intensity *Φ* (*v*_1_, *v*_2_) in synchronous spectra shows the same symbol (both negative or both positive) as the correlation peak *Ψ* (*v*_1_, *v*_2_) in asynchronous spectra, the change of band *v*_1_ is prior to or earlier than that of band *v*_2_, and vice versa.^[189]^ Besides, if the correlation intensity in synchronous spectra is not zero (or blank), but zero in asynchronous one, then the movements of bands at *v*_1_ and *v*_2_ are simultaneous. Noda's rules are summarized as follows: (1) If *Ф*(*v*_1_, *v*_2_) > 0, *Ψ*(*v*_1_, *v*_2_) > 0 or *Ф*(*v*_1_, *v*_2_) < 0, *Ψ*(*v*_1_, *v*_2_) < 0, the change at *v*_1_ takes place prior to or earlier than that at *v*_2_. (2) If *Ф*(*v*_1_, *v*_2_) > 0, *Ψ*(*v*_1_, *v*_2_) < 0 or *Ф*(*v*_1_, *v*_2_) < 0, *Ψ*(*v*_1_, *v*_2_) > 0, the change at *v*_2_ takes place prior to or earlier than that at *v*_1_. (3) If *Ф*(*v*_1_, *v*_2_) > 0, *Ψ*(*v*_1_, *v*_2_) = 0 or *Ф*(*v*_1_, *v*_2_) < 0, *Ψ*(*v*_1_, *v*_2_) = 0, the change of *v*_1_ and *v*_2_ are simultaneous.

### 4.4 Typical interaction between polymer and nano units

After understanding the interaction mechanism between polymer and solvent, the general rule of polymer swelling could be roughly inferred. Then, if the PC structure is integrated into such a LSP (Figure 4c), the migration of these micro‐nano particles could be driven by the swelling of the polymer network.[^27^, ^190^] The corresponding parameters in the Bragg equation can be changed, thus producing variational structural color. Conversely, the change of color indirectly feedbacks the response of the polymer to the external stimulus and then visualizes or even quantifies the stimulus source. This color change comes from the physical quantity change of PC and does not involve the interference of other new substances. Therefore, SPCSs are particularly suited to play a role in some interdisciplinary disciplines, such as drug patches with built‐in color sensing,^[191]^ porous catalytic carriers,^[192]^ and so on.

The interaction force (chemical bonds or physical entanglements) between the polymer and PC structure is the key to the color change of PCs.^[28]^ In a typical ionic elastomer PC, 2D correlation analysis of temperature‐dependent FTIR spectra suggests that chemical associations (H‐bonds and ion‐dipole interaction‐formed lithium bonds) exist among the polymer chains, ionic liquid, and PC structural units (Figure 4d).^[159]^ Li^+^ acts as the physical crosslinking agent of the polymer chain through the action of lithium bonds, thus improving the mechanical properties of elastomer PC. The correlational analysis at the molecular level inspires revealing the sensing mechanism of polymer‐based PCs.

The diffusion of solvent between polymer and nano‐unit indirectly regulates the swelling of polymer and the migration behavior of nano‐unit. For example, in a typical electroresponsive polymer‐opal composite PC, the electrolyte diffusion needs to reach the electrode surface across the entire thickness, existing a greater resistance to reach equilibrium swelling.^[193]^ In an inverse opal PC, the macropores of orderly interconnection reduce the diffusion length of electrons and ions into and out of the polymer gel necessary for maximum expansion of the lattice. The compatibility of the polymer with the nano unit is the primary condition for the change of the lattice response to the stimulus,^[80]^ otherwise it is very likely to result in phase separation and loss of sensing performance.[^52^, ^194^] The interaction between the two could be controlled by regulating the aggregation of nano units.^[195]^ It mainly affects the linear tensile modulus and dissipated energy of the material. Some functional groups (such as silicon hydroxyl) on the surface of nano units can produce H‐bonds and van der Waals forces with polar groups between polymer molecular chains (other functional groups and polymers might also exist dipole‐dipole, ion‐dipole, etc.).[^143^, ^169^] During the response of some SPCSs, in addition to the structural factors, these interaction forces might be another key factor affecting the lattice change in SPCSs. The birth of LSP‐based SPCSs directly avoids the design and preparation of specific molecular probes. Its flexible color‐changing mode and recyclability make it develop rapidly in the field of smart sensing. Next, this review will specifically introduce the corresponding LSP‐based SPCSs according to the PC dimension.

## 5 STRUCTURAL DESIGN AND FUNCTIONAL APPLICATION OF LSP‐BASED SPCSs

Depending on the working environment, the structural design of SPCSs and the way it is combined with polymers are varied. Different PC structures show different advantages, for example, the combination of rigid opal PC and polymer can avoid the swelling of the microsphere itself, the inverse opal can provide a richer specific surface area, etc. The reasonable choice of polymer can also improve the related sensing properties. Filling the prepared SPCS with a new polymer can often improve the response time or corresponding effectiveness. The output of structural colors could be controlled through geometric modifications at the micron scale or larger,[^196^, ^197^, ^198^, ^199^] especially the angle independence. In conclusion, polymer screening and reasonable PC structure design are the keys to preparing high‐performance SPCSs.

### 5.1 Typical LSP‐based 1D SPCSs

**FIGURE 5:**
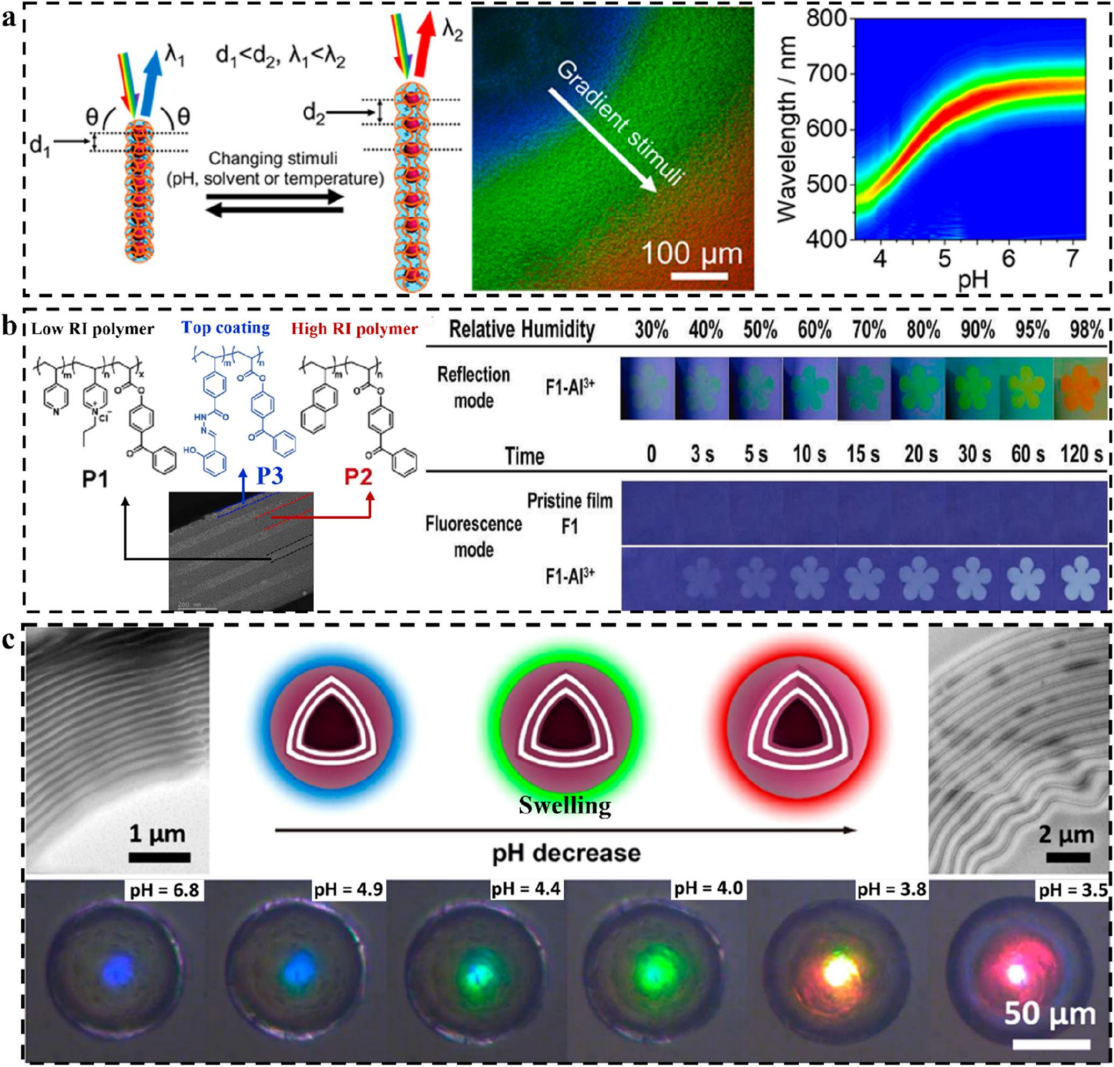
(a) Color‐changing mechanism and the corresponding pH sensing of the magnetic responsive 1D nanochains, Copyright 2020,^[206]^ American Chemical Society; (b) Layered stack structure of dual‐responsive 1D SPCS and corresponding structural color and chemical color sensing,^[207]^ Copyright 2023, Elsevier; (c) Swelling diagram and pH sensing of concentric‐layered block copolymer microcapsules,^[208]^ Copyright 2020, American Chemical Society.

LSP‐based 1D SPCSs are composed of LSP and 1D PCs, the polymer can be either a structural component of the PC or can be used to wrap or hold the PC.[^200^, ^201^] It usually depends on the specific response of the polymer to change the relevant parameters of the PC, thus producing variational structural colors.[^202^, ^203^] The response way of 1D SPCS formed by the combination of rigid microspheres and polymers can be either the active movement of the microsphere or the indirect movement caused by the swelling/shrinking of the polymer. The most typical example is magnetic microspheres. The lattice spacing and particle ordering of 1D PCs can be manipulated by magnetic fields. The polymer may consist of several especially responsive materials, such as pH‐responsive,^[125]^ humidity,^[204]^ and thermoresponsive^[205]^ polymer hydrogels. The hydrogel wraps the magnetically induced regular arrangement of microspheres to form nanochains. Under neutral conditions, the hydrogel is in swelling equilibrium and the rigid microspheres are arranged in a constant state. Once the external pH changes, the swelling equilibrium of the hydrogel shifts, resulting in volume expansion or shrinkage, which indirectly leads the microsphere to move (changing the lattice spacing). The most prominent result is the change in the structural color of the system. Guan and collaborators prepared hydrogel‐based photonic nanochains using the H‐bonds guided template polymerization method to achieve high‐resolution real‐time pH detection of the reaction solution (Figure 5a).^[206]^ The pH induces the elongation of the nanochain, indirectly changing the lattice spacing of 1D PC, thus feedback on the structural color information. By changing the types of polymers (e.g. introducing different functional groups), various stimuli‐responsive photonic nanochains could be designed and prepared. This type of 1D SPCS is generally stored in a liquid environment. The nanochains in a swelling equilibrium state shows strong topography switching characteristics, which is especially suitable for smart sensing in a liquid environment.

1D SPCSs can be fabricated by stacking rigid nanoparticles and polymers layer on layer.[^99^, ^209^] During the response, the polymer (as the low‐refractive‐index unit) produces swelling or shrinking through the interaction with the solvent, driving the nanoparticles (as the high‐refractive‐index unit) to move and expand the layer spacing, resulting in structural color changing.^[210]^ The rigid layers can also be considered as swelling sites.[^211^, ^212^] The corresponding stimulus can be deduced from the structural color and the reflection wavelength. During this period, polymer molecules or some functional groups can also bind specifically to the stimulus, resulting in specific optical phenomena. The associated chemical bonding processes often exhibit reversibility, demonstrating the recyclability of smart molecular materials. By using the specific complexation of thiourea and amide with Hg^2+^, Ma *et al.* induced the contraction of the low‐refractive‐index layer by intermolecular force of the polymer layer, realizing the visual sensing of Hg^2+^ based on the change of the layer spacing.^[213]^ The high‐density sulfur recognition sites in 1D SPCSs can capture and enrich Hg^2+^ in large quantities through chelation, triggering the change of structural color. Enriched Hg^2+^ could be desorbed under the action of dilute hydrochloric acid, which is of great significance for the clean recovery of Hg^2+^. Polymers with different refractive indices can also be assembled to form 1D SPCS by alternating stacking. By introducing chemical color materials in the outermost layer, dual mode sensing of structural color and chemical color can also be realized (Figure 5b). Jung and Lee *et al.* prepared 1D PC by alternating deposition of photo crosslinked poly (2‐vinylnaphthalene‐co‐benzophenone acrylate) and 51%‐quaternized poly (4‐vinylpyridine‐co‐benzophenone acrylate).^[207]^ A dual‐responsive 1D SPCS was obtained after coating poly ((2‐hydroxybenzylidene)‐4‐vinylbenzohydrazide‐co‐benzophenonyl acrylate). Dual color sensing was achieved by water‐induced swelling of low refractive index layers and the fluorescence enhancement induced by the coordination of Al^3+^.

1D SPCSs can also exist in the form of eccentric or concentric circles.[^214^, ^215^] The latter can produce angle‐independent structural color with significantly enhanced monochromaticity due to its high symmetry. Such 1D microstructure is particularly useful in structural color sensing because it does not need to take into account incident or observation angles. Xu and Zhu *et al.* constructed concentric‐layered block copolymer microcapsules through a combination of self‐emulsification and emulsion‐solvent evaporation, whose structural color varied with the thickness and refractive index of the core layer (Figure 5c).^[208]^ This 1D SPCS can be swollen in ethanol or an acidic solution to change the lattice spacing, thus displaying a changing structural color. The special structure of the microcapsule only allows the surface concentric layer to swell and shrink within a limited range. The drug release behavior can be observed by using the structural color, which has important significance in the field of pH sensing and programmed drug loading and release.

The most remarkable advantage of 1D SPCSs is that the synthesis and self‐assembly of nanoparticles are relatively simple, and the related sensors can be obtained quickly through the alternating spin coating process. There is almost no special requirement for the compatibility of high and low refractive index materials, which allows designers to have more selectivity and collocation in the selection of materials. In the reflection spectra, it can also use its second or even third‐order diffraction.^[211]^ Therefore, the swelling layer of 1D SPCSs allows a large degree of volume variation. Based on its material change in one dimension, the patterning modification of the PC is also particularly easy. 1D SPCSs composed of nanochains are also suitable for most kinetics monitoring involving liquid chemical reactions, which is much more convenient than other PC materials. Carefully designed center‐symmetric or axisymmetric 1D SPCS particles or fibers can also compensate for structural color sensing errors caused by the corresponding angle dependence. However, the reflection peaks of 1D SPCSs typically display a wide half‐peak width,^[99]^ limiting their recognition in the field of structural color sensing. Future studies urgently need to address this shortcoming.

### 5.2 Typical LSP‐based 2D SPCSs

2D SPCSs are easier to fabricate than 1D and 3D PCs because only single‐layer assembled micro‐nano arrays are required.[^173^, ^216^] The pioneers of such sensors are Asher and co‐workers.^[100]^ They elucidated the corresponding diffraction and sensing mechanisms. The 2D array could be transferred to the surface of the polymer substrate and fixed by chemical or physical action.[^217^, ^218^] Once the substrate has undergone a physical change (such as swelling, shrinking, sunken, bending, etc.),^[197]^ the Bragg parameters of the 2D PC (lattice spacing, incident angle, etc.)^[219]^ will change, resulting in the changing of structural color.^[220]^ Single‐layer arrays are much easier to change than 1D and 3D arrays.^[221]^ At the same time, the change of lattice spacing can be quantified by the change of the Debye diffraction ring.[^222^, ^223^] Therefore, 2D SPCS shows a pivotal position in the field of analytical sensing.

**FIGURE 6:**
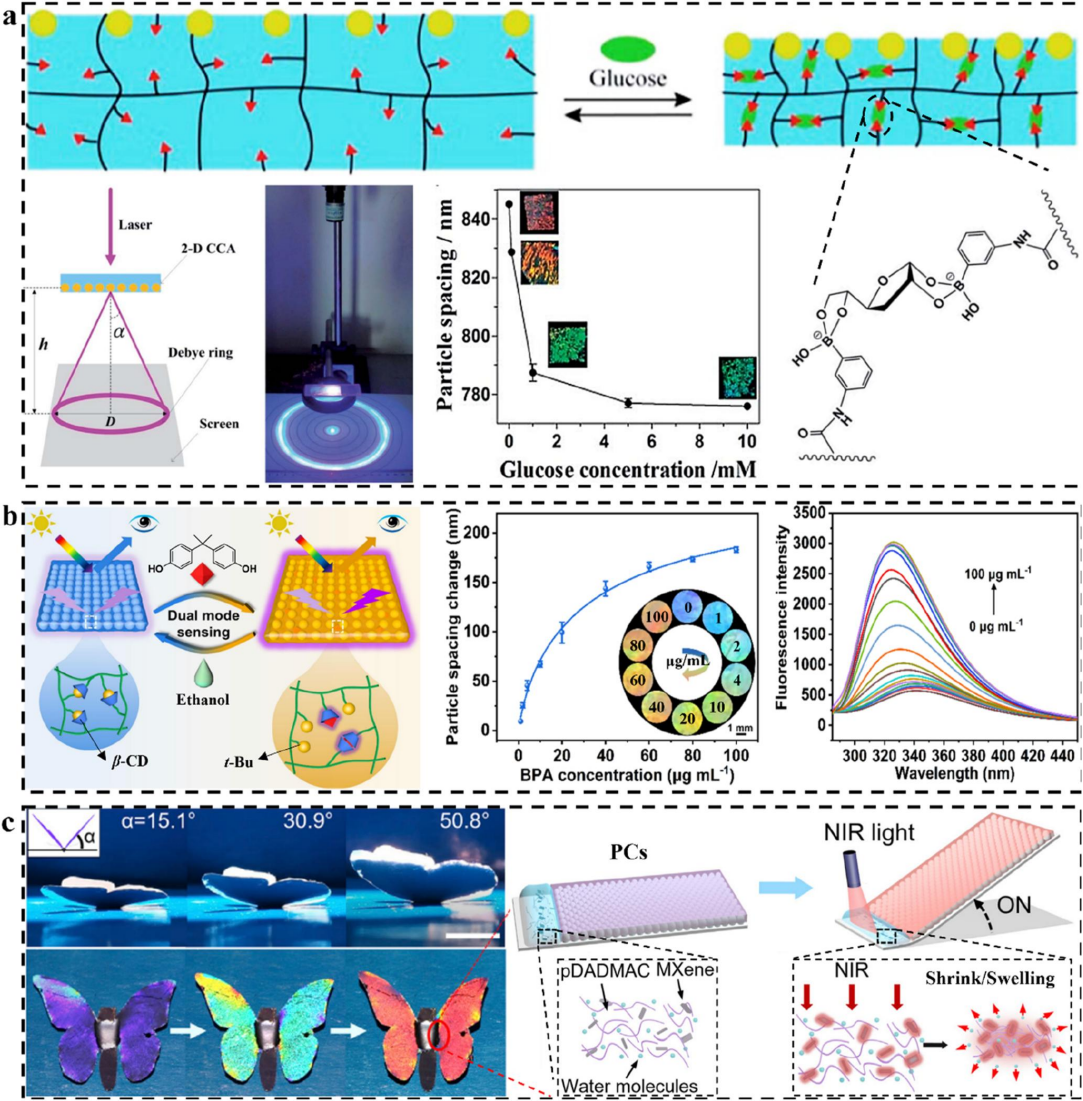
(a) Phenylboric‐modified 2D SPCS and corresponding mechanism for selective sensing of glucose,^[227]^ Copyright 2014, Royal Society of Chemistry; (b) Host‐guest interaction based 2D SPCS for colorimetric and fluorescent dual‐mode sensing of bisphenol A,^[228]^ Copyright 2023, American Chemical Society; (c) Dynamic structural color soft actuator based on the light‐induced swelling/shrinking,^[229]^ Copyright 2022, Elsevier.

By designing the type of polymer or introducing specific responsive groups/molecular imprinting, 2D SPCSs can also produce specific responses to specific stimuli.[^100^, ^224^, ^225^] Taking the phenylboric group as an example,^[103]^ due to its affinity for diol‐containing molecules, polymer chains could be easily cross‐linked by specific molecules (such as glucose).^[226]^ The interaction between them will result in a change in the volume of the polymer system. Therefore, such molecules or groups are often considered for the construction of 2D SPCSs to detect molecules containing o‐diol structures. Meng and Xue *et al.* prepared 2D SPCS for visual sensing of glucose by attaching 2D PC to the surface of a phenylboric‐modified polymer hydrogel (Figure 6a).^[227]^ The combination of glucose with two adjacent phenylboric acids increases the crosslinking density of the polymer hydrogel, causing the system to shrink, thereby increasing the Debye diffraction ring diameter. At a physiological ion intensity of 150 mM, 2D SPCS shows significant sensitivity for glucose in the whole physiological glucose range of the human body, providing inspiration for the monitoring of human physiological information.

Based on the sensitivity and detection limits of 2D SPCS comparable to fluorescent probes, the combination of structural color materials and fluorescent molecules provides a more reliable analytical method for the detection of stimulus. The colorimetric sensing of structural color and the change of fluorescence intensity can simultaneously support the accuracy of the analysis results. The combination of two coloration materials is inseparable from the bridge of polymer molecules. On the one hand, the polymer carries the PC arrays. On the other hand, the emission intensity is regulated by the bonding between the internal groups and the fluorescent molecules. By using the host‐guest interaction of β‐cyclodextrin (β‐CD) and tert‐butyl (t‐Bu), Xiao *et al.* designed the dynamic cross‐linked molecular structures and constructed 2D SPCS for colorimetric and fluorescent dual‐mode sensing of bisphenol A (BPA) (Figure 6b).^[228]^ As a competitive guest, BPA breaks the balance of the host‐guest interaction between β‐CD and t‐Bu and unties the interchain crosslinking, thereby reducing the cross‐linking density of the polymer and triggering the swelling of the polymer. The increase of lattice spacing of 2D SPCS results in structural color change. Moreover, based on the Debye diffraction ring diameter or structural color, the sensitive and selective detection of BPA could be achieved with a detection limit of 1 μg mL^−1^. The competitive formation of β‐CD/BPA host‐guest significantly enhanced the intrinsic fluorescence of BPA, and the corresponding detection limit reached 0.001 μg mL^−1^. Structural color and fluorescence sensing share the same reaction, resulting in a wider dynamic response range than single‐signal sensing strategies. This 2D SPCS is a typical case of the combination of PCs and fluorescence molecules, and it is worth popularizing in the field of related analysis and detection.

Another prominent feature of 2D PCs is the angle‐dependence of the structural colors.^[230]^ It is different from the mirror angle correlation of 1D and 3D PCs, 2D PCs can observe bright structural colors over a very wide range of angles, and the color changes with the incident and observation angles.^[231]^ Therefore, if the angle of the 2D PC plane is changed, the structural color can also be changed. This feature makes 2D PCs play important roles in color sensing in the field of soft actuators, especially surfaces involving 3D spatial motion. Li *et al.* integrated a photothermal responsive hydrogel (so‐called artificial muscle) and 2D PC to construct a 2D SPCS‐based photoresponsive soft actuator with structural color self‐reporting (Figure 6c).^[229]^ Under near‐infrared irradiation, MXene rapidly converts light energy into heat. The water diffusion and reversible hydration/dehydration within the material causes the artificial muscle to swell/shrink, triggering the fanning of the 2D SPCS plane. The change of angle results in structural color change. The triggering conditions for this type of 2D SPCSs could be designed according to the type of artificial muscle materials, which inspires further development of self‐driven PC devices that respond to light, electronic or magnetic fields, temperature, or chemical signals.

The advantages of 2D SPCSs are the simple way for construction of PC array, rapid response of structural color, and high detection sensitivity. The adhesion of the single‐layer array to the substrate can hold the 2D PC with high fastness and without considering the array disturbance caused by polymer filling. The volume change of 2D SPCSs is due to the swelling and shrinking of the substrate, so it is easy to achieve a specific response to the stimulus source. Many smart sensors could be made by designing special functional groups of polymers. 2D PC sensors have attracted more and more attention because of their advantages such as convenient preparation, low cost, simple detection requirements, high accuracy, and good periodicity after introducing recognition molecules.^[232]^ It should be noted that 2D PCs usually utilize its out‐of‐plane diffraction and Debye rings. The observed structural colors show a strong angle‐dependence, which is both an advantage and a disadvantage depending on the detection conditions. For example, during *Candida albicans* sensing,^[77]^ strong angle‐dependence on structural color interferes with color changes, but in 3D shape changes, it is excellent for real‐time visualization of changes in the internal stress of the material.^[217]^ Reasonable use of angle‐dependent structural colors is an important strategy for future visualization sensing of related 2D SPCSs.

### 5.3 Typical LSP‐based 3D SPCSs

3D SPCSs are structural color materials composed of LSPs and 3D PCs. The corresponding coloration and color change mechanisms are similar to those of 1D and 2D SPCSs. The difference is that the artificial design of the 3D SPCS structure is more diverse. Therefore, 3D SPCSs are currently the most widely developed and used structural color materials. Back in 1997, Asher and co‐workers broke through the absorption spectrum‐based traditional sensing and first reported the 3D SPCS based on a colloidal array and polymer gel, presenting a new sensing strategy through the diffraction changes of PC.^[233]^ The swelling of such a 3D SPCS was realized by the specific binding of polymer to metal ions and the consequent change of osmotic pressure, outputting variational structural color. Since then, a large number of SPCSs have been developed.[^11^, ^48^, ^130^, ^234^] Due to the adjustable lattice spacing, only a limited swelling can meet the maximum reflection wavelength of PC in the visible range. To prevent excessive swelling of the system, usually, LSPs can meet the material requirements of the relevant SPCSs. Depending on the working conditions, specific swelling can be achieved by designing chemical groups or molecular structures.

#### 5.3.1 Common structural design strategy of 3D SPCSs

**FIGURE 7:**
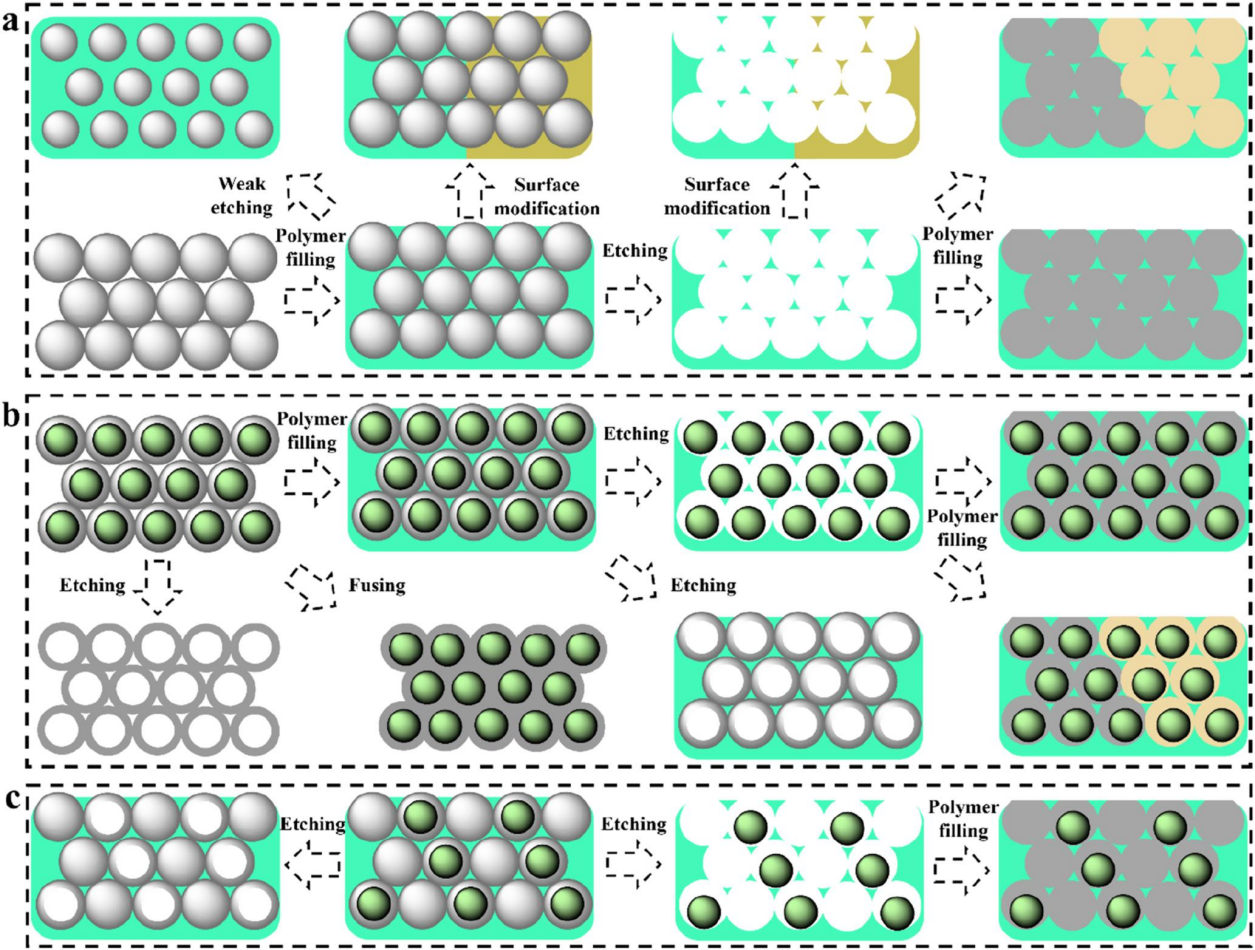
(a) Opal PC formed by self‐assembly of homogeneous microspheres and corresponding derived PC structures; (b) Opal PC formed by self‐assembly of core‐shell microspheres and corresponding derived PC structures; (c) Opal PC formed by self‐assembly of homogeneous and core‐shell microspheres and corresponding derived PC structures.

The coloration units used to fabricate 3D SPCSs generally include solid microspheres, core‐shell microspheres, hollow spheres, inverse opals, double inverse opals, and their combinations.^[235]^ The LSPs can be a component of the coloration unit, or they can be a filling material or a substrate material. The most common coloration units are opals and inverse opals. Opal PC templates can be obtained by the ordered self‐assembly of homogeneous microspheres (Figure 7a). The corresponding composite films can be obtained by filling special responsive polymers. The so‐called structural color sensing of these films is dominated by changes in lattice spacing induced by polymer swelling. The homogeneous microspheres can also be etched or incompletely etched^[236]^ to obtain corresponding inverse opal PCs or composite films with reduced particle sizes. The polymer of the obtained composite film can also be secondary modified,^[237]^ such as hydrophilic modification^[114]^ and crosslinking density. Similarly, the inverse opal PCs can also be secondary modified or filled with other polymers to form new SPCSs. The chemical or physical properties of these filling materials can alter the swelling properties of the system, thereby regulating the associated structural color sensing.

The SPCS systems constructed of core‐shell microspheres have various structural control modes, and the objects to be etched include the core and shell.^[238]^ For the opal PCs obtained by self‐assembly of core‐shell microspheres, hollow‐sphere‐type PCs can be obtained after removing the core (Figure 7b).[^238^, ^239^] For soft shells, at high temperatures (greater than the glass transition temperature), the polymer materials of the shell can fuse with each other to form a more mechanically stable PC film.^[240]^ Similarly, these PCs could be filled with other polymers to form SPCSs with special responsiveness.[^241^, ^242^] The shell and core of SPCSs can also be selectively etched to form anisotropic SPCSs. The shell‐removed SPCSs are the so‐called double inverse opal PCs, and the embedded microspheres can move freely in the macropore space. It has been shown that the coloration of those structural color materials is related to the arrangement of the embedded microspheres, and the coloration rate is controlled by the speed of the formation of the regular arrangement of microspheres. The etched SPCSs can also be filled with polymers. The obtained SPCSs will display different photophysical properties. For example, by filling with hydrophilic acrylamide, the color rendering speed of SPCSs could be greatly accelerated after solvent stimulation. Core‐shell microspheres can also be assembled with homogeneous microspheres in a set sequence to obtain anisotropic composite SPCS films (Figure 7c). Both the core and shell could be selectively etched and further filled with other polymers. Take ZnS@SiO_2_ core‐shell microsphere PC as an example, hydrofluoric acid can etch the shell (SiO_2_) and retain the core, while hydrochloric acid can etch the core (ZnS) and retain the shell.^[243]^ The coloration mechanism of structural color materials is much the same. Reasonable design of PC structure is the key to investigating the corresponding sensing properties. Recognizing and understanding them can bring convenience to the design of smart sensors.

#### 5.3.2 Typical opal‐derived SPCSs

**FIGURE 8:**
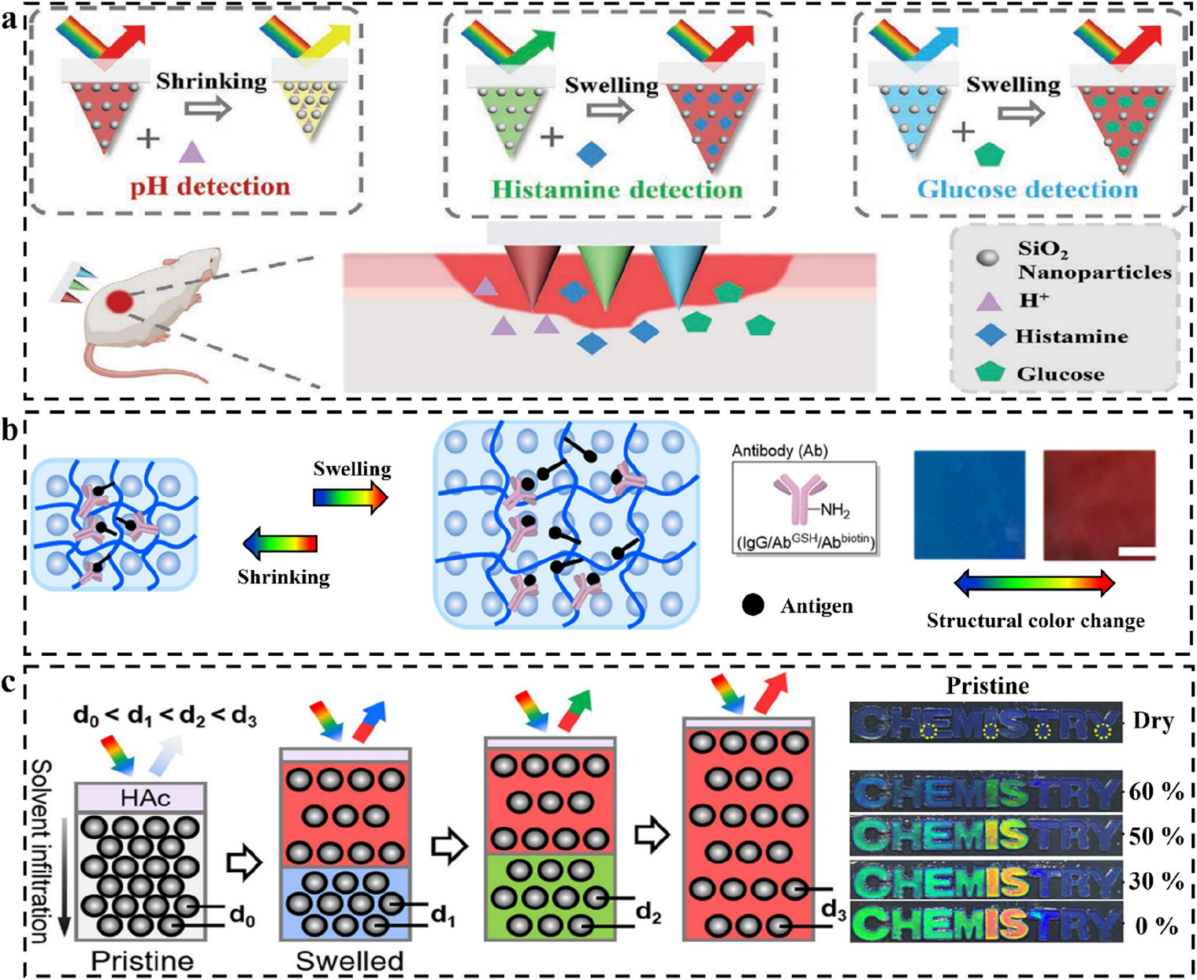
SPCSs derived from opal PCs. (a) The structural color microneedle patches with partitioned and layered SPCSs for wound small molecules sensing,^[246]^ Copyright 2023, Wiley‐VCH; (b) Universal PC biosensors based on the antibody‐antigen interactions,^[247]^ Copyright 2020, American Chemical Society; (c) Nonuniform swelling diagram of SPCS and corresponding structural color in ethanol with different concentration,^[248]^ Copyright 2021, Elsevier.

Structural color sensing of polymer‐based opal SPCSs introduced in this section mainly depends on the isotropic and nonuniform swelling of the polymers. PCs as a face‐centered cubic structural material with PBGs are composed of monodisperse colloidal nanoparticles assembled in an orderly manner.[^124^, ^244^] Non‐close‐packed PCs can fuse with stimulus‐responsive polymers, expanding or contracting in response to stimuli, resulting in shifts in the spectra and even visual changes in structural color.^[245]^ As described earlier, swelling is the chain movement of polymer molecular chains due to the uniform diffusion and adsorption of solvents. The PC structure moves passively due to the interchain binding forces, resulting in the change of lattice spacing and structural color. By designing specific binding sites, many specific responsive SPCSs based on swelling have been widely developed. Zhao *et al.* combined PCs and microneedle arrays to develop structurally colored microneedle patches for multiple wound biomarker detection (Figure 8a).^[246]^ Different SPCS modules were constructed by using a partitioned and layered casting strategy. Due to their small dimension and high specific surface area, SPCSs can extract and enrich many biomarkers in the skin's interstitial fluid, thereby detecting them, including pH (based on the interaction between hydrogen ions and carboxyl groups), glucose (reaction of o‐diol groups with fluobenzoborate), and histamine (specific recognition of aptamers and target molecules). The swelling/shrinking‐induced volume change of the three sensing modules under the stimulus of the target molecule leads to the structural color change and characteristic peak shift of PCs, and the target molecule could be qualitatively measured by spectral analysis.

In addition to traditional solvent‐induced swelling/shrinking, Wang and Yao *et al.* recently reported a non‐solvent‐induced swelling behavior (Figure 8b).^[247]^ Using antibody‐antigen‐specific interactions, the first competition‐based biological SPCS capable of detecting various biomolecules (such as proteins, peptides, and small molecules) with the naked eye was reported. The non‐covalent “cross‐linked” network of antibodies and antigens causes SPCSs to be in a “contracted” state. When the SPCS is exposed to the analyte, the exchange between the analyte and the fixed antigen dissociates the non‐covalent “cross‐linked” network, resulting in a significant but reversible expansion. The reflection wavelength shift generated by swelling is entirely dependent on antibody‐antigen interactions. This study has broken the traditional swelling‐induced color change of PC. Antiboy‐antigen competitive SPCSs show important application value in the field of bioenzyme detection and disease diagnosis.

Nonuniform swelling is generally caused by the inhomogeneous diffusion and adsorption of solvents in the polymer and is also related to the cross‐linking density. In a “solvent‐solvent” diffusion system, the properties of the solvents play important roles in the swelling behavior of the polymer. Materials with similar Hansen solubility parameters show high affinity with each other, which is conducive to swelling and dissolution of polymers in solvents. Yang and Huang *et al.* regulated the affinity between the polymer and the solvent by changing the crosslinking density and proposed the nonuniform swelling mechanism of SPCSs according to the difference in the diffusion distance of the solvent along the direction perpendicular to the surface of the PC film (Figure 8c).^[248]^ For SPCS with a certain thickness, due to the limitation of solvent diffusion distance and time, the expansion of the bottom layer on the lattice spacing is limited. The corresponding swelling could be divided into rapid swelling of the top layer, solvent transport to the bottom layer, and slow swelling of the bottom layer. Accordingly, a series of solvent‐sensing strategies are proposed. For example, in the acetic acid‐ethanol system, depending on the inhibition effect of ethanol on the polymer affinity, the swelling of the polymer induced by ethanol with different concentrations can produce different shifts of reflection. The corresponding ethanol concentration could be inversely deduced from the reflection wavelength. On this basis, the time‐dependent dynamic reflection spectra generated by solvent diffusion and exchange can also be used to detect solvents and even isomers.

The opal‐derived SPCSs have no special requirements for the stiffness and toughness of the polymer. They could be fabricated by filling the opal PC templates with polymers, or by polymerizing liquid PC obtained from “metastable” assembly.[^56^, ^249^] Considering the solubility of monomers to some microspheres (such as polystyrene), coating with silica would be better. The disadvantage of opal‐derived SPCSs is that it is difficult for the stimulus source to spread quickly throughout the PC system, which results in a slower equilibrium response time. Fortunately, researchers can use such equilibrium time to identify some compounds, or even homologs,^[116]^ through the DRS of the diffusion process. We believe that different PC architectures must be significant in the history of SPCS.

#### 5.3.3 Typical inverse opal‐derived SPCSs

**FIGURE 9:**
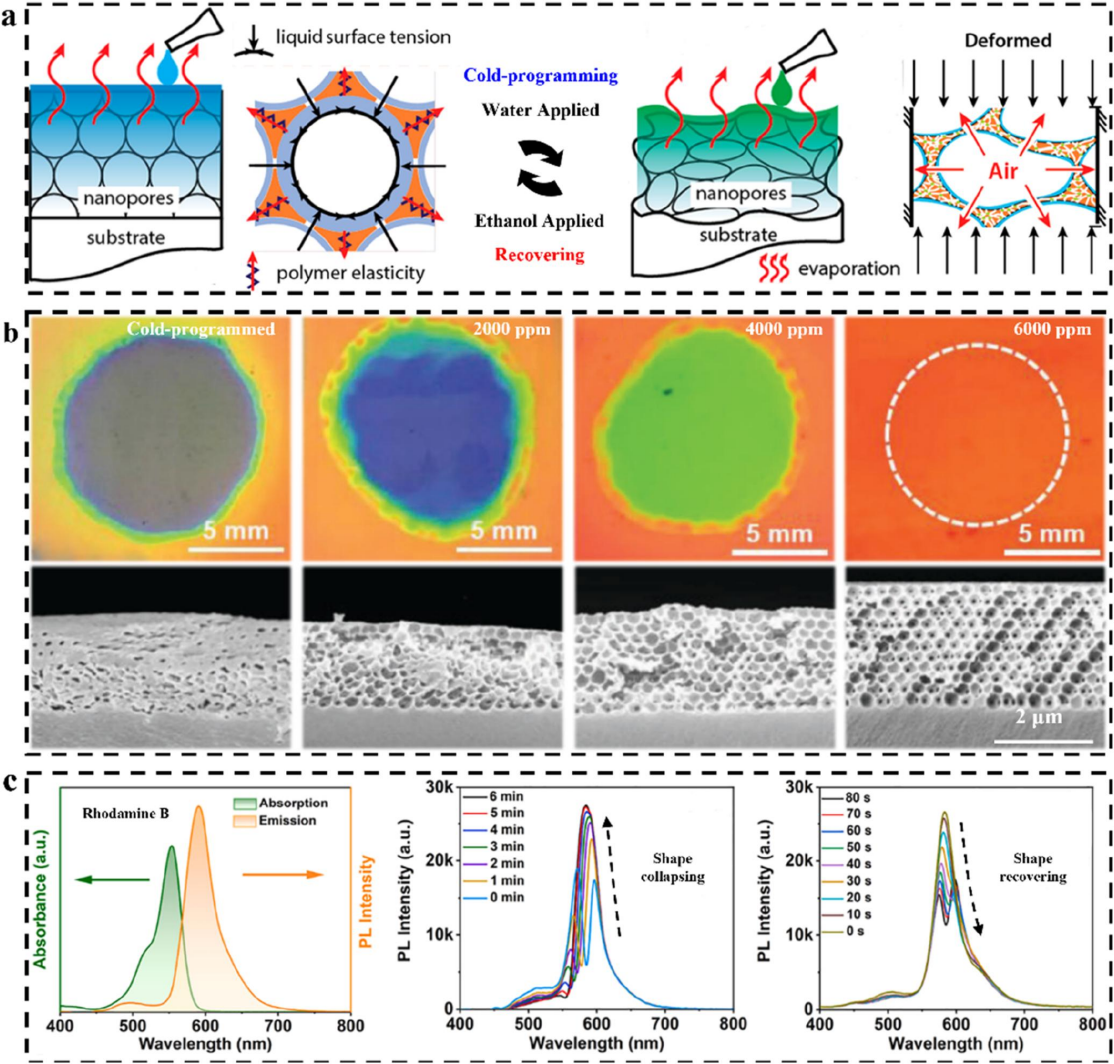
SPCSs derived from inverse opal PCs. (a) Swelling‐induced unconventional shape memory mechanisms of SPCSs,^[250]^ Copyright 2018, American Chemical Society; (b) The stepwise shape recovery of the collapsed macroporous of SPCSs,^[117]^ Copyright 2018, Wiley‐VCH; (c) Reversible regulation of photoluminescence of shape memory SPCSs,^[251]^ Copyright 2022, Elsevier.

The swelling of inverse opal SPCSs generally includes anisotropic and isotropic swelling. Inverse opal macropores undergo deformation during swelling, including collapse, diameter increase or decrease, and skeleton wall thickening or thinning.^[28]^ The most typical example is the shape memory PC (Figure 9a).^[250]^ The main driving force is capillary pressure.^[252]^ After wetting by solvent, on the one hand, the solvent molecules diffuse and adsorb to the polymer chains, unbinding the interchain and accelerating the molecular chain motion. On the other hand, solvent wetting and swelling soften the polymer skeleton, making it more susceptible to being deformed. The disordered collapse and ordered recovery of the macropores are determined by the elastic modulus of the polymer skeleton and the capillary pressure generated by solvent evaporation.^[108]^ Different from swelling results of other PCs, the PBG of inverse opal SPCSs will disappear or blue shift due to the irregular collapse of macropores, reappear due to the shape recovery of macropores, redshift due to the increase of macropore size, blue shift due to the increase of skeleton wall thickness, etc.[^253^, ^254^] On the one hand, the change of macropore can produce a change of structural color, on the other hand, the change of micro‐nano size of macropores directly regulates the corresponding wetting behavior.[^177^, ^255^] Color changes are often associated with these changes in performance. Moreover, the structural changes of the macropore channels are reversible, all thanks to the swelling of the polymer materials.^[112]^ Therefore, a series of physical or chemical properties of inverse opal SPCSs can be regulated on demand by using SMPs. Macropore shape memory based on polymer swelling will provide ways for structural optimization of porous materials. The corresponding light manipulation is of guiding significance to the design and construction of SPCSs.

Based on the swelling‐induced stepwise shape recovery of inverse opals, Jiang and Taylor *et al.* designed highly sensitive and specific SPCSs that can selectively detect many swelling analytes (such as ethanol, acetone, and dichloromethane) in non‐swelling solvents (such as gasoline and water) (Figure 9b).^[117]^ Swelling causes a sharp decrease in glass‐transition temperature and elastic modulus of the polymer, and the strong capillary pressure generated by solvent evaporation forces the macropore deformation. When collapsed SPCSs are exposed to a mixture of liquids or vapors containing both swelling (e.g., ethanol) and non‐swelling (e.g., alkanes and water) solvents, the concentration and time‐dependent diffusion of the swelling analytes leads to a gradual shape recovery of the inverse opal macropores. Depending on the concentration of the analyte, each intermediate state displays a specific diffraction color which could be used as a corresponding colorimetric indicator. Similar macropore shape changes can also be combined with fluorescent molecules. The fluorescence intensity could be regulated by the PBG.[^123^, ^256^, ^257^] By combining Rhodamine B into shape memory PCs, Zhang and Zhu *et al.* realized the recovery of fluorescence intensity through the collapse of macropores and the reduction/enhancement of fluorescence intensity through the solvent swelling‐induced recovery of macropores (Figure 9c).^[251]^ The corresponding regulation could be attributed to the matching of the photon band gap with the emission of Rhodamine B. We believe that this mutual coordination of structural and chemical colors will provide inspiration for the development of analytical methods and will promote the development of related SPCSs.

**FIGURE 10:**
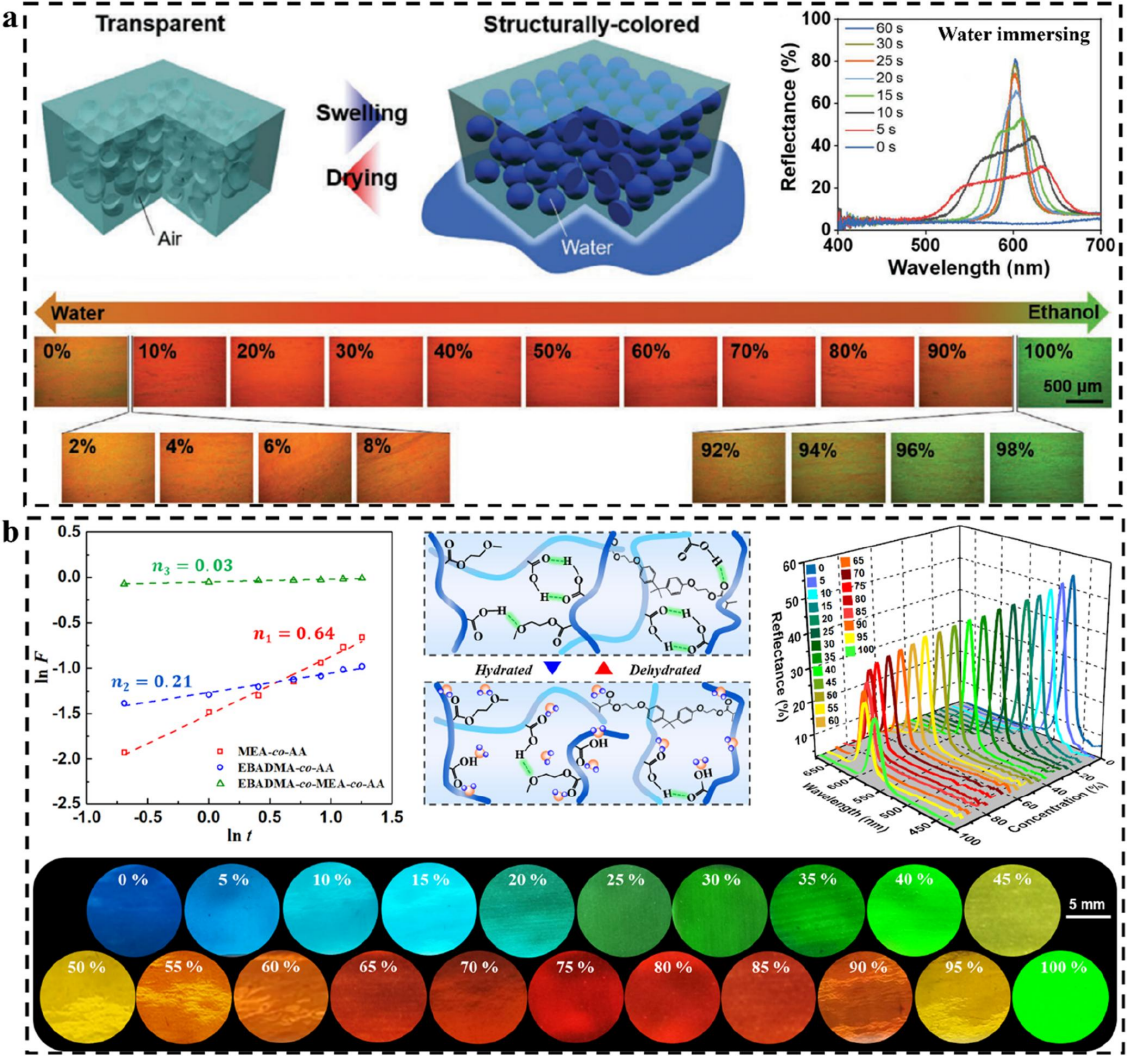
(a) Reversible switching between transparent and structural color states, coloration process after water immersing, and ethanol concentration sensing of inverse opal SPCS,^[111]^ Copyright 2020, Wiley‐VCH; (b) The swelling kinetics curves, mechanism, and ethanol concentration sensing of colorimetric inverse opal SPCS,^[154]^ Copyright 2023, Elsevier.

The swelling of inverse opal SPCSs regulates the morphology of macropores.^[258]^ The swelling degree is generally controlled by polymer crosslinking density and interchain bonding force. For hydrophilic polymers with low elastic modulus, in the dry state, the inverse opal macropores generally show a collapsing state, displaying a transparent state. Under the action of a solvent (such as water), the collapsed macropores can recover to their ordered shape due to the polymer network swelling, showing the structural color. With the increase of swelling degree, the morphologies of macropores continue to change, which further changes the lattice spacing and structural color. Kim *et al.* observed nonuniform swelling of inverse opal macropores in SPCS constructed of polyethylene glycol diacrylate (Figure 10a).^[111]^ The reflection at the initial swelling stage shows wide peaks of low intensity, which can be attributed to the excessive expansion of the surface lattice and the restricted swelling of the inner lattice. A narrow peak with high intensity at swelling equilibrium could be attributed to the swelling equilibrium of the inner and outer lattices of the inverse opal. This nonuniform swelling was derived from the fact that the diffusion of water in recovered macropores is faster than that in close‐packed collapse structures. Depending on the affinity of the polyethylene glycol chain to water (higher than ethanol) and acrylate crosslinking junctions to ethanol (higher than water), changing the content of both can regulate the swelling of SPCS in water or ethanol. At the same time, for a set ratio, the prepared SPCS showed different swelling in different ratios of the ethanol‐water mixture, to show different structural colors. The modification of the associated swelling by regulating the affinity between the polymer and the solvent inspires the design of SPCS at the molecular level.

Moreover, the swelling of macropores could be further regulated by changing the bonding force among polymer chains, especially the chemical bonds with weak bonding forces, such as H‐bonds. On the one hand, those chemical bonds can produce interchain bonding forces, on the other hand, they can be recombined under the action of solvent or external force, giving the polymer chain more adjustable motion. Based on the Fickian diffusion model, Zhang *et al.* developed a portable colorimetric SPCS with confined swelling according to the reconstruction of H‐bonds among molecular chains (Figure 10b).^[154]^ The swelling exponent of the copolymer in water (∼0.03 < 0.5) indicates that SPCS has a stable swelling equilibrium in solution. According to the affinity between the solvent molecules and the polymer chains, SPCS showed different degrees of swelling in different concentrations of ethanol solutions. The morphology and size of macropores changed with the change in ethanol concentration. The structural color corresponding to each concentration remains stable, which proves that this SPCS is particularly suitable for ethanol concentration sensing. In addition, the real‐time diffusion of the ethanol‐water mixture and the co‐solvency effect of ethanol‐water on copolymer could be tracked through structural color. Benefiting from the swelling induced by copolymer H‐bond reconfiguration, this kind of SPCS has important implications in the fields of alcohol distillation, drug release, compressure sensing, chemical engineering analysis, molecular tracking, etc.

As the most common weak chemical bonds, H‐bonds can not only restrain the movement of polymer chains through intermolecular bonding force but also form self‐sealing structures to block the contact between polymer chains and stimulus. For example, the self‐sealing effect of H‐bonds forces the hydrophilic material to produce water resistance. The effect of weak chemical bonds on the swelling properties of polymers can be further enhanced and functionalized by the asymmetric transformation of micro‐nano structures, such as selective swelling. On the one hand, the reconstruction of H‐bonds changes the external affinity, on the other hand, regulates the interchain bonding force of different parts. In collapsed porous structures, changes in the wetting threshold usually produce anisotropic wetting. The interchain H‐bonds combined with the change of micro‐nano structures will inevitably affect the corresponding physical properties of porous materials.

**FIGURE 11:**
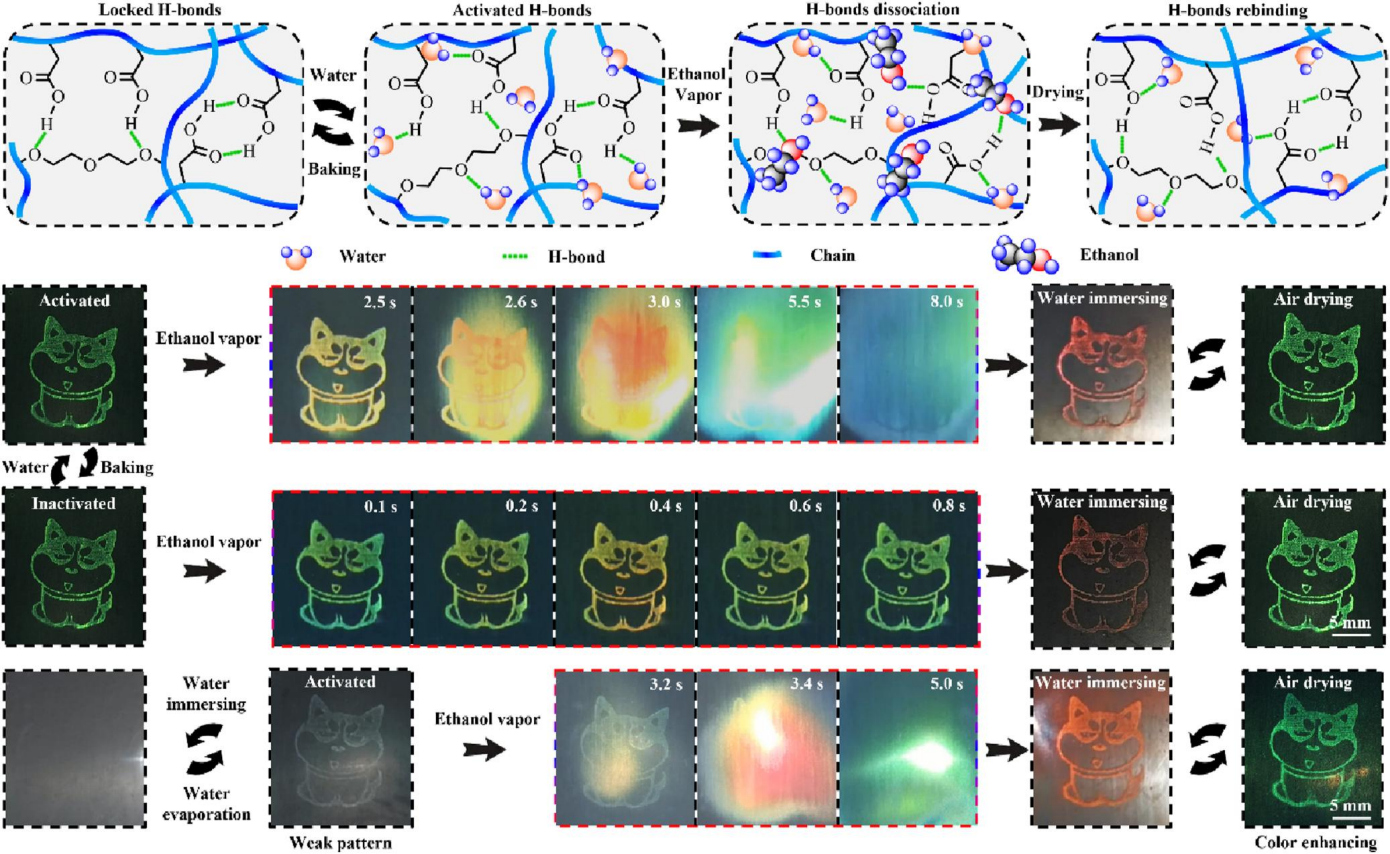
The ethanol vapor‐responsive mechanism of activatable SPCS at the molecular level and the dual‐confined shape‐memory effects of structurally colored patterns,^[109]^ Copyright 2023, Wiley‐VCH.

Zhang *et al.* observed anomalous structural color changes in shape memory PCs by reconstructing interchain H‐bonds (Figure 11).^[109]^ Only water‐wetted inverse opals exhibit a color response to ethanol vapor. Water molecules and polymer chains form temporary active sites through H‐bonding to capture ethanol produced by capillary condensation, and then the swelling effect of ethanol triggers the release of the entropy elasticity in polymer chains, resulting in the shape recovery of the collapsed macropores. In addition, water can selectively activate pressure‐coded SPCS through H‐bond reconstruction. The activated region could be re‐coded (confined response) in ethanol vapor. Undergoing water evaporation, the re‐coding pattern disappears, displaying the initial pattern. Importantly, the weak structural color pattern was enhanced by undergoing ethanol vapor‐induced shape recovery and water evaporation‐induced anisotropic collapse. All these indicate that the swelling properties of the polymer are not only related to the affinity of the material to the solvent but also to the geometry of the micro‐nano structures. The birth of activatable responsive structural color materials will promote the expansion of nanophotonics in the fields of water printing, erasable watermarks, signal amplifiers, information coding, and selective sensing.

**FIGURE 12:**
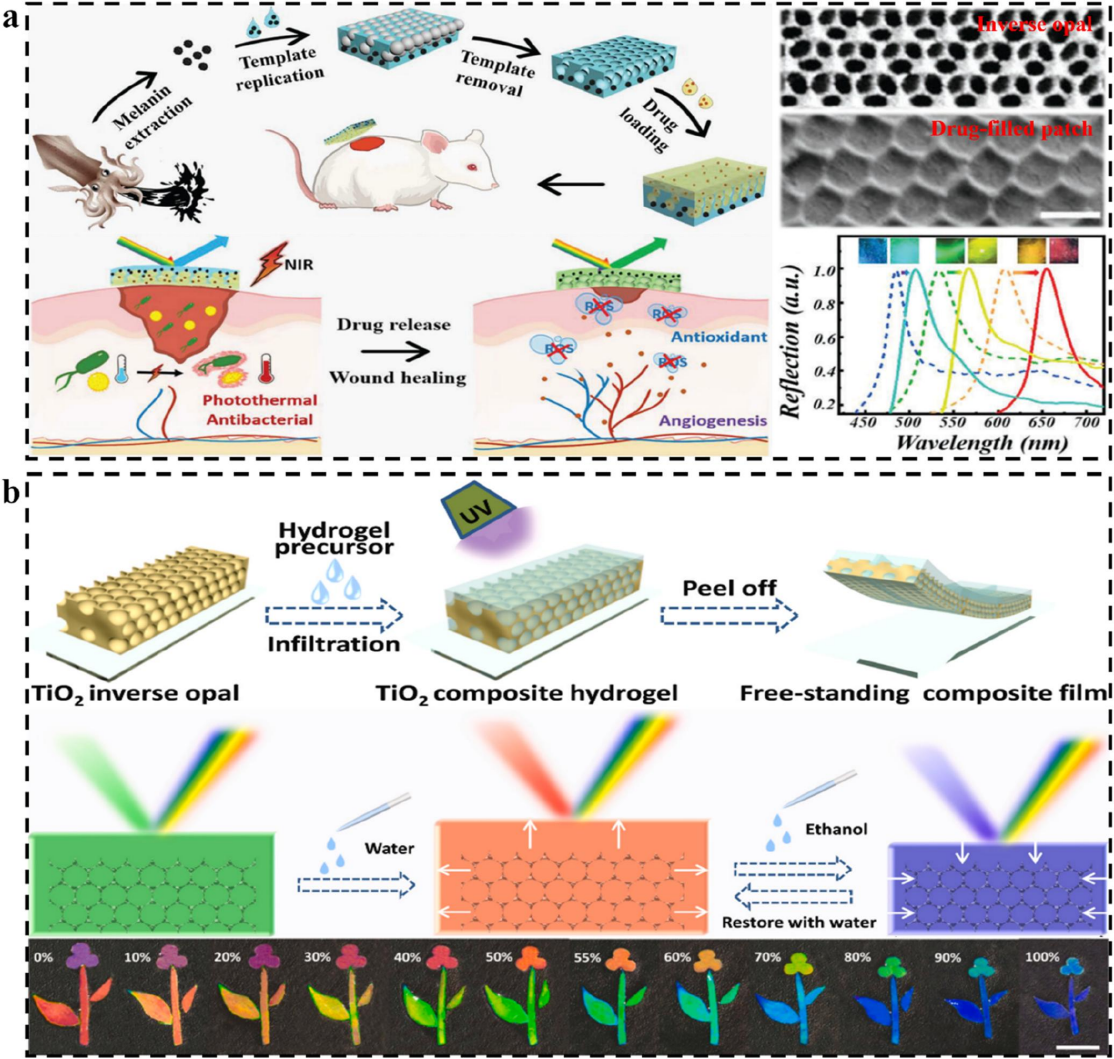
SPCSs derived from inverse opal PCs. (a) Preparation of inverse opal SPCS patches, working principle for promoting bacteria‐infected wound healing, and corresponding structural color sensing,^[191]^ Copyright 2023, Wiley‐VCH; (b) Schematic diagrams of the fabrication of the TiO_2_ inverse opal composite SPCS and the corresponding structural color sensing of ethanol solutions with different concentrations,^[259]^ Copyright 2021, Elsevier.

The macropores of the inverse opal SPCS can also be filled with other polymer materials. The sensing of such SPCS could be regulated by the physical or chemical properties of the filling materials. The functions of the PCs and the filling materials can be both independent and interrelated. Therefore, some interdisciplinary applications could be realized through the filling and fusion of materials. For example, filling a PC with hydrogels rich in medically active drugs can bridge drug therapy with structural color sensing. Zhao *et al.* constructed an inverted opal skeleton by using fish gelatin methylacrylyl pregel containing melanin nanoparticles and then filled with asiatic acid‐mixed agarose pregel to construct an SPCS hydrogel patch with bacteria‐infected wound healing and structural color sensing (Figure 12a).^[191]^ On the one hand, melanin nanoparticles promote agarose liquefaction and release asiatic acid through photothermal effects, killing bacteria and promoting wound healing. On the other hand, the light absorption of melanin enhances the structural color of PC and improves color recognition. With the release of asiatic acid, the *n*_eff_ changes, resulting in the change of structural color and reflection wavelength. Thus, the drug release could be measured simply by detecting the wavelength shift of the SPCS hydrogel patch, and monitoring corresponding delivery processes. SPCS hydrogel patch provides important reference values for the application of polymer‐filled PCs in drug delivery, soft robotics, healthcare monitoring, biosensing, and other fields.

Inverse opal SPCSs can also be composed of inorganic materials, especially metal oxide materials with high refractive index, such as titanium dioxide (TiO_2_),^[260]^ tin dioxide (SnO_2_),^[261]^ and tungsten trioxide (WO_3_).^[21]^ A high refractive index can usually improve the reflection intensity of PC and enhance the structural color. On the one hand, the filled polymer enhances the stability of the inverse opals, on the other hand, it can give some specific stimulus responses to the system. The design principle of this type of SPCS is similar to that of opal‐polymer composite films. The difference is that the polymer filling rate of inverse opal is greater than that of opal. For example, in a typical FCC structure, the porosity of the inverse opal (∼0.74) is greater than that of the opal (∼0.26), which makes the inverse opal more susceptible to the modulation of the filled polymer.

By using polyacrylamide‐based TiO_2_ reverse opal PC hydrogels modified with PEGDA and 2‐acrylamide‐2‐methylpropyl sulfonic acid (AMPS), Chen *et al.* investigated the visual detection of different solvents and ethanol solutions with different concentrations based on the high refractive index difference between TiO_2_ and the filling material and the swelling difference of the system to different solvents (Figure 12b).^[259]^ The high refractive index difference between TiO_2_ and the filled polymer provides a bright structural color for the composite SPCS. The hydrophilicity of SPCS is enhanced by the sulfonic acid group of AMPS, and the crosslinking degree is regulated by PEGDA. All these promote the effective swelling of the system in response to solvent stimulation and thus realize the color sensing with a wide wavelength span. In addition, the design of multi‐module microchips makes it possible to integrate composite SPCS based on TiO_2_ inverse opal with computers, accelerating the development of PCs in the field of smart sensing.

Inverse opal‐derived SPCSs inherit the 3D structure of the opal templates, and the significant advantage is the rich porous channel, which is conducive to the diffusion of stimulus sources in the whole PC system in a short time. As a result, the associated response time is short. Inverse opal polymers are essentially porous materials, and the connected macropores are particularly suitable for the design and construction of micro‐reactors due to the outstanding mass transfer.[^21^, ^192^, ^262^, ^263^] The properties of inverse opal‐derived SPCSs mainly depend on the chemical and physical properties of the polymer skeleton. They are generally films with good mechanical properties. Those macropores could be isotropic or multilayered structures.^[264]^ Through reasonable template design, the inverse opal constructed can also cover the front and back of the film.^[26]^ Therefore, the functional design of inverse opal‐derived SPCSs generally starts from the selection of polymer materials. Inverse opals can also achieve specific responses by filling with other polymers or by patterning modification. Given the diversity of structural design and material selection, we believe that the future construction of SPCSs will still give birth to different types of inverse opal‐derived SPCSs.

#### 5.3.4 Typical core‐shell microsphere‐derived SPCSs

**FIGURE 13:**
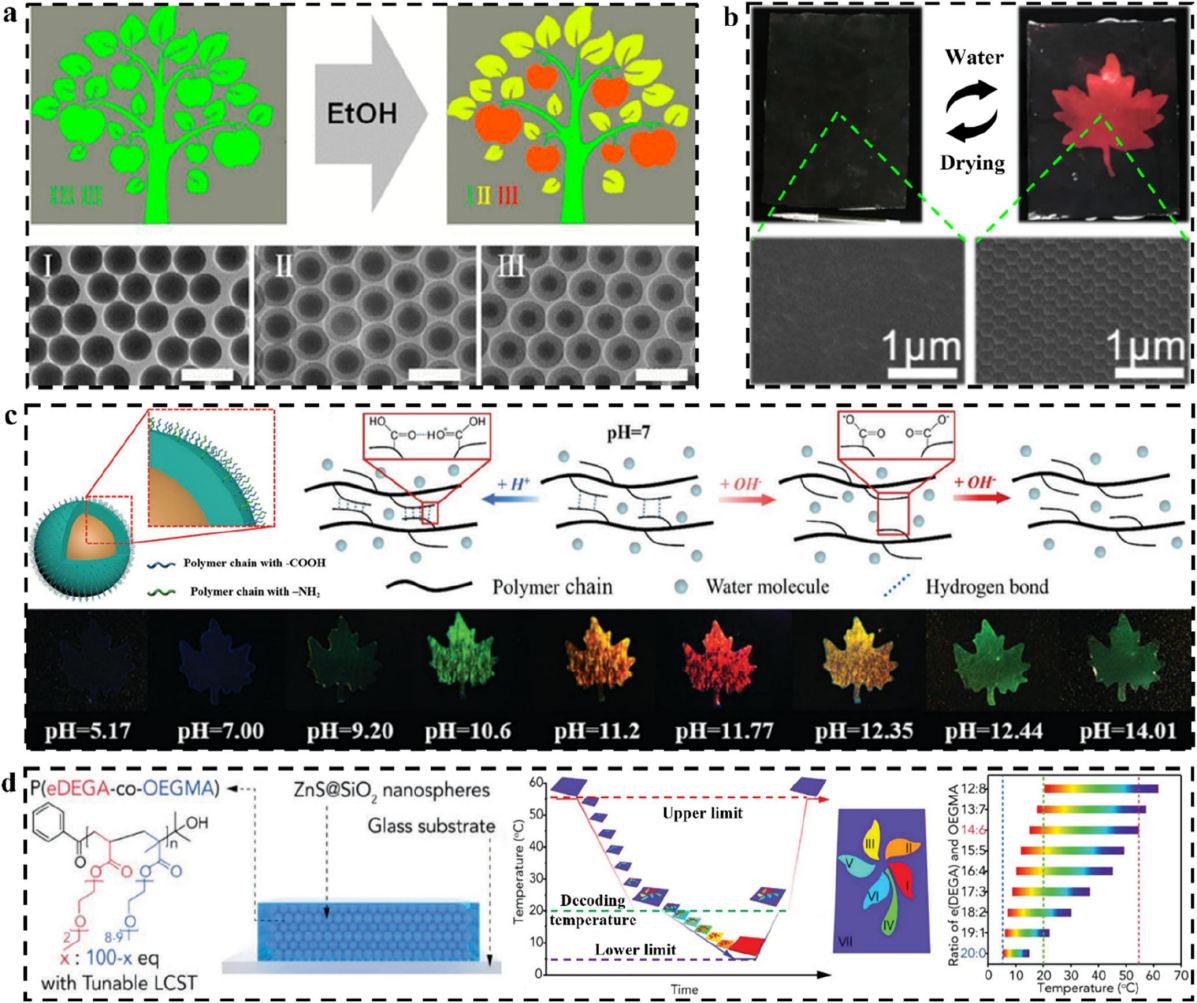
Core‐shell microsphere PCs and derived SPCSs. (a) Typical core‐shell microsphere‐based PC and its ethanol vapor response,^[265]^ Copyright 2014, American Chemical Society; (b) Water rewritable PC films and corresponding change of micro‐nano structures before and after response,^[266]^ Copyright 2019, American Chemical Society; (c) Ph‐induced swelling mechanism and corresponding structural color sensing of the core‐shell microsphere‐based SPCS,^[125]^ Copyright 2019, Wiley‐VCH; (d) LSP‐filled SiO_2_‐coated ZnS core‐shell microsphere‐based SPCS and corresponding temperature sensing,^[36]^ Copyright 2023, Wiley‐VCH.

Core‐shell microspheres are generally composed of a core and a shell of different materials. The overall size could be controlled by the thickness of the shell. The core could be rigid materials, magnetic nanoparticles, polymers, or other high‐refractive‐index metal oxides. According to the Bragg equation, the core‐shell microspheres with different particle sizes can be programmed to obtain PCs with similar structural color by adjusting the size of the core and shell. Structural color sensing could be realized by using the differential response of the shell. For example, Gu and collaborators constructed core‐shell microsphere‐based SPCSs with the same structural color but different vapor responses by simultaneously controlling the solid core and mesoporous shell thickness of microspheres (Figure 13a).^[265]^ Based on the change of *n*_eff_ of PCs caused by capillary condensation, the Bragg diffraction displacement of PCs with the same core but a thicker mesoporous shell or the same diameter but a higher mesoporous rate can be larger.

In contrast, swellable core‐shell microspheres usually consist of a rigid core and a soft shell. The swelling effect is usually exerted by the shell, usually composed of LSPs.^[267]^ Such kind of polymers generally show a low glass transition temperature, which could be fused shell to shell by heating.^[268]^ Sometimes there is no special requirement for the material of the cores. Such kind of core‐shell microspheres can even be prepared by emulsion polymerization at one time. This kind of core and shell of the microsphere are generally formed spontaneously by phase separation or preferential polymerization. For example, Zhang *et al.* constructed water‐rewritable PC films with large areas through the air‐liquid interface self‐assembly method (Figure 13b).^[266]^ Through the fusion of the outer layer by heating, the ordered microspheres lose their PC structural characteristics and become transparent. Once in contact with water, the hydrophilic outer polymer separates due to the movement of the polymer chains, re‐forming PC structures, increasing the *n*_eff_ of the system, and thus displaying structural color.

Zhu and Gu *et al.* also constructed responsive elastic poly (methyl methacrylate‐butyl acrylate) copolymer SPCS using core‐shell microspheres (Figure 13c).^[125]^ In addition to the temporary disappearance of PC structure caused by shell fusion and the structural color reappearance triggered by water wetting, this SPCS also produces different swelling in different pH solutions. High swelling will enlarge the refractive index difference between the shell and core and increase the lattice spacing, thus output redshifted and enhanced structural color. With the further increase of pH, the deprotonated carboxyl groups will produce high osmotic pressure, resulting in a decrease in lattice spacing, thus outputting blue‐shifted structural colors. Benefiting from the fusion and separation of micro‐nano structures and reversible swelling of shells, core‐shell microsphere‐based SPCSs provide an epoch‐making inspiration for the rapid construction of structural color sensors with large areas.

For PCs formed by self‐assembly of rigid core‐shell microspheres, swelling SPCSs could be constructed by polymer filling. The associated coloration and color change mechanisms are similar to those of opal‐polymer composite SPCSs. The difference is that the core of such core‐shell microspheres could be nanoparticle clusters or irregular nanoparticles that are difficult to self‐assemble in order. Usually, they have a higher refractive index to compensate for the irregularity of the materials. These cores might be polymers or metal oxides. Core‐shell microspheres with regular morphology could be obtained by coating them with other materials that could be molded easily. Moreover, the overall refractive index of the microsphere can be designed by the sizes of the core and shell. With the help of the surface chemical and physical properties of the coated shell, the self‐assembly efficiency of the microspheres could be greatly improved, thus obtaining the corresponding opal PC templates.

Similarly, depending on the working conditions and purpose of use, core‐shell microsphere composite SPCSs could be obtained by filling some functional polymers. The types of filled polymers are variable. The filled areas could be patterned (including patterned arrays and patterned surface modifications, etc.). For example, Wu *et al.* demonstrated programmable thermochromic SPCSs by filling thermally responsive polyethylene glycol acrylate copolymers in SiO_2_‐coated ZnS core‐shell microsphere opal PCs (Figure 13d).^[36]^ The responsive temperature range of SPCSs was manipulated by adjusting the monomer ratio. The color change mechanism of SPCSs is due to the change of lattice spacing induced by polymer swelling at different temperatures. Based on this, the SPCSs prepared by mask patterning display similar structural colors under normal conditions. The target structural color pattern was programmed only in an aqueous solution at a specific temperature. This programmed structural color display points to a new direction for traditional anti‐counterfeiting of PCs and inspires precision temperature sensing.

**FIGURE 14:**
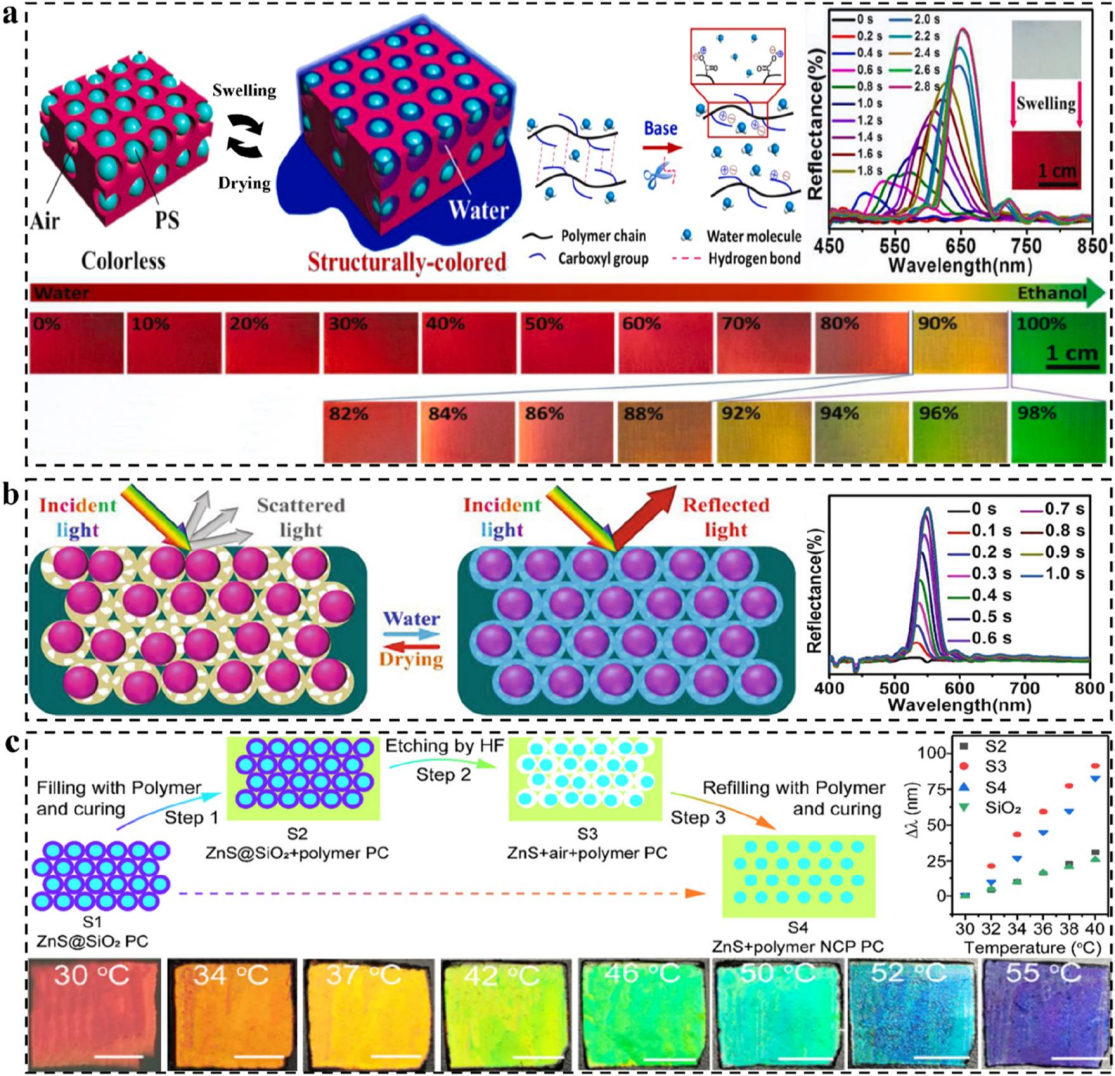
SPCSs derived from core‐shell microsphere PCs. (a) The swelling and coloration of the double‐inverse opal SPCS and corresponding structural color sensing of ethanol solutions,^[43]^ Copyright 2021, Elsevier; (b) The coloration mechanism and corresponding real‐time reflection of the polymer‐filled double‐inverse opal SPCS during water response,^[269]^ Copyright 2022, Elsevier; (c) The preparation of nonclose‐packed SPCS, the thermal response and structural color sensing of each PCs,^[270]^ Copyright 2021, American Chemical Society.

After etching the shell of the opal‐polymer composite PC assembled by the core‐shell microspheres, the inverse opal PC embedded microspheres could be obtained, also known as the double inverse opal PC. After the removal of the shell, the pores allow the core to be randomly arranged. Due to the light scattering effect, the double inverse opal PCs generally show no obvious structural color in the dry state. The corresponding double inverse opal immediately displays structural color after solvent wetting. Taking PS@SiO_2_ as an example, when the obtained inverse opals embedded with PS microspheres are wetted by solvents (such as water), the randomly distributed PS microspheres will rearrange by buoyancy and surface tension, producing Bragg reflection and bright structural color. The inverse opal skeleton limits the random movement of PS microspheres. On the other hand, the type of skeleton material can also be adjusted according to the working conditions to produce some specific responses. Zhang *et al.* inhibited the formation of H‐bonds between polymer chains and enhanced the hydrophilicity of SPCS by deprotonation of carboxyl groups in the polymer skeleton via saturated sodium carbonate solution (Figure 14a).^[43]^ Water as a good solvent of the modified polymer can promote the swelling of the skeleton, while ethanol as a bad solvent can inhibit the related swelling, thus manipulating the lattice spacing of the SPCS. Therefore, the modified SPCS can produce different reflections and structural colors when dealing with ethanol solutions with different concentrations, which shows the potential of ethanol concentration sensing, especially the accurate colorimetric sensing of ethanol with high concentrations.

Subsequently, they filled the pores of the double inverse opal PC with polyacrylamide hydrogel, which greatly improved the hydrophilicity of the SPCS, thereby shortening the coloration time from ∼2.4 to ∼0.8 s (Figure 14b).^[269]^ The reason is that the hydrophilic polyacrylamide hydrogel accelerates the penetration and diffusion of water and promotes the swelling of the polymer chain, resulting in a rapid ordered rearrangement of the built‐in randomly distributed PS microspheres, and thus producing bright structural colors. Polymer‐filled double inverse opal PC provides a method for fast sensing of SPCS.

The artificial design of the PC structure gives these materials flexible and changeable light manipulation properties. Some metal oxides with high refractive index can also be embedded in inverse opal macropores by the above method. It is usually difficult for some pure oxides to self‐assemble to form regular and orderly opal PC templates. By coating SiO_2_, it can obtain self‐assembly properties similar to SiO_2_ microspheres. After filling with polymer materials, the composite film with high refractive index metal oxide microspheres or clusters was obtained by etching the shell. Similarly, SPCSs with specific functions could be obtained by further filling the pores with polymers.

Wu *et al.* prepared SPCSs with the thermal response and mechanochromic by filling poly‐N‐isopropyl acrylamide (PNIPAm) hydrogel into the macropore of the inverse opal embedded with zinc sulfide (ZnS) microspheres (Figure 14c).^[270]^ The resulting nonclose‐packed PC structures show ample room for the regulation of particle movement when the system volume changes. The filled PNIPAm‐based hydrogel will swell due to water absorption or shrink due to dehydration, resulting in a change in the lattice spacing of the PCs. When the temperature rises from 30°C to 55°C or under 20% compressive strain, the maximum reflection of SPCS shows a blue shift of more than 200 nm, showing a sensitive structural color change. The SPCSs constructed by using the polymer‐filling method inspire the construction of non‐close‐packed PC structures and the design of smart sensors based on volume changes to produce structural color changes.

In addition to opal‐derived SPCS‐like properties, the most interesting aspect of core‐shell microsphere‐derived SPCSs is the double‐inverse opal PC,^[271]^ which is a structure of inverse opal embedded with microspheres. Unlike the inverse opal PC, the optical properties of the double inverse opal are not only related to the polymer skeleton but also regulated by the ordered distribution of the embedded microspheres. Furthermore, the core‐shell structure has inspired researchers to construct non‐close‐packed SPCSs by removing the coated shell and then refilling polymers.^[270]^ At the same time, it provides methods to assemble anisotropic microspheres. The shells of core‐shell microsphere‐derived SPCSs can be some kind of soft polymer. More structural changes could be achieved by heating or hot pressing. The removal and retention of the shell and core and the selective filling of the polymer will generate new micro/nanostructures, giving rise to a variety of structural color materials.

### 5.4 Swelling‐induced angle‐dependent structural color sensing of SPCSs

**FIGURE 15:**
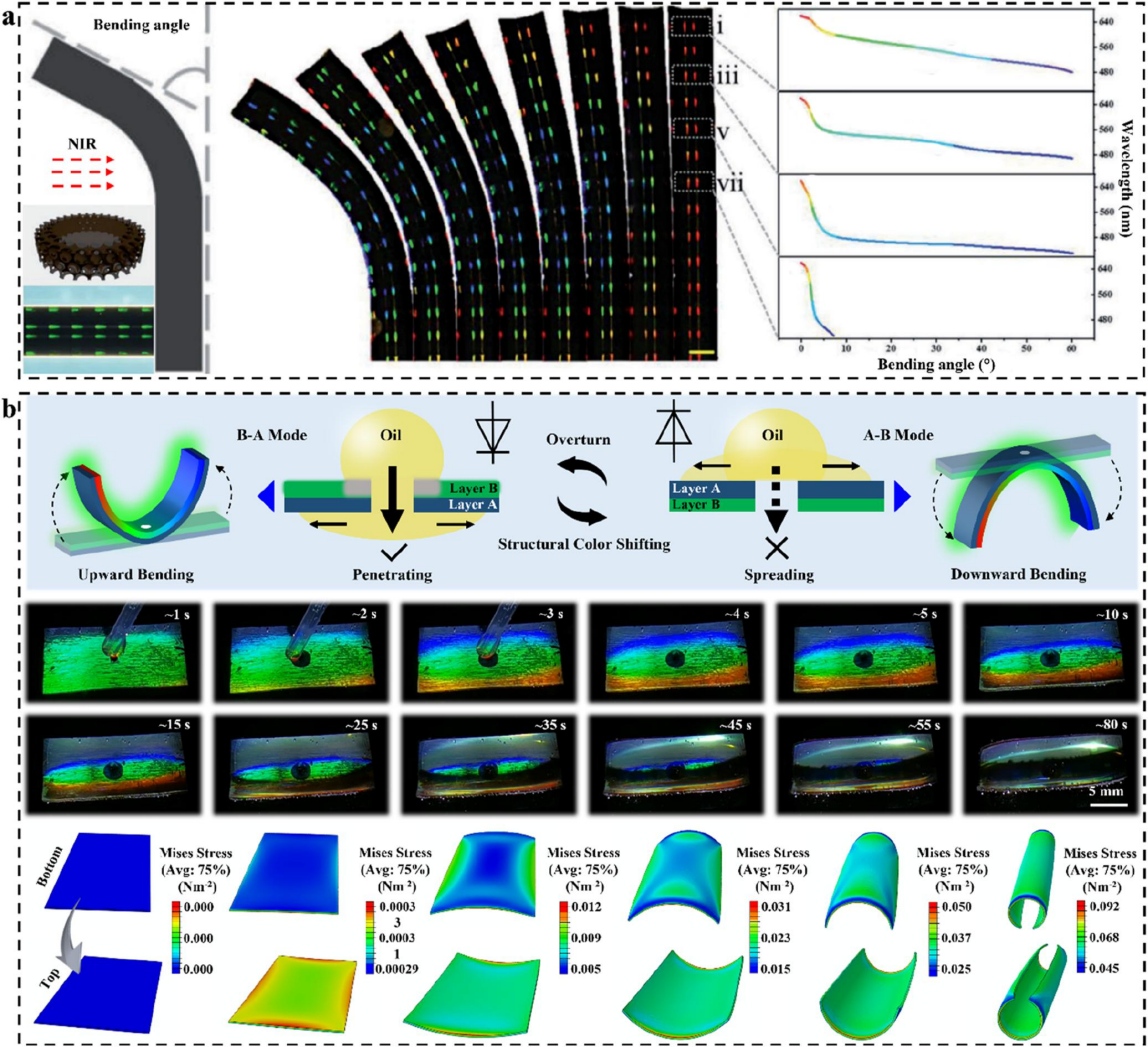
Swelling‐induced angle‐dependent structural color. (a) Light‐controlled reversible bending of structural color stripes with self‐reporting color indication,^[272]^ Copyright 2017, Wiley‐VCH; (b) Asymmetrical swelling‐induced bending of Janus photonic soft actuator and corresponding structural color sensing of internal stress,^[45]^ Copyright 2022, Royal Society of Chemistry.

According to Bragg's law, the structural color of PCs is generally angle‐dependent. For common PCs, the structural color shows a blueshift with the increasing incident angle. Therefore, changes in angle can interfere with the structural color sensing of the SPCSs. The default detection condition of structural color sensing of most PCs is vertical incidence (that is, both the incident and the detection angle are 0°). In 2D PCs, diffracted light scatters from a wide range of angles, and the related structural color sensing is also susceptible to angle. In 3D PCs, the common reflection usually occurs in the mirror angle. Therefore, once the PC plane is bent, the initial observation point is likely to miss the structural color.^[92]^ However, structural color sensing of some PCs utilizes angle‐dependence. The stimulus‐induced bending behavior of the PC plane is one of the ways to correlate angle and stimulus source. Due to the influence of the substrate, the bending of the system can change two Bragg parameters, including the lattice spacing and the incident/detection angle. In the structurally colored soft SPCS designed by Zhao and Gu *et al.*, the photothermal effect induces the soft stripe to shrink (one‐sided shrinkage that could be attributed to the swelling and shrinking of PNIPAm hydrogel due to its temperature‐induced hydrophilic to hydrophobic transition), resulting in phototropic bending and extrusion of inverse opal structures (Figure 15a).^[272]^ The shrinkage causes the lattice spacing to decrease, which results in the blueshift of structural color. Although this structural color change cannot be attributed to the incident angle, it establishes the correlation between structural color and bending angle, providing a new way for the development of smart sensors involving bending behavior. The most typical application is the visual detection of biological cell activity and related drug activity.

Some 3D PCs display angle‐dependent structural colors similar to 2D PCS on the same side of the light source. The entire film displays iridescence during bending. The color of each area is determined by incident and detection angles, both of which are modulated by the bending angle. Therefore, any stimulus behavior that can produce changes in structural color due to the angle changes of the PC plane could be fed back by structural colors. The bending of the film is usually caused by asymmetric swelling,^[273]^ which could be regulated by setting asymmetric wettability or gradient crosslinking. For example, by integrating an underwater super‐oleophilic copper micro‐nano array and oil‐phobic inverse opal PC, Zhang *et al.* reported a soft bilayer photonic actuator based on the Laplacian pressure difference‐controlled diode‐like unidirectional transport of underwater oil droplets (Figure 15b).^[45]^ The difference in oil‐induced swelling between the super‐oleophilic layer and the oil‐phobic layer leads to the directional bending of the film. According to the angle‐dependent retroreflection of 3D PCs (similar to the light diffraction and scattering of 2D PCs), the real‐time internal stress distribution during film bending was revealed by structural color. The change of structural color was matched with the finite element simulation of the internal stress. This angle‐dependent retroreflection inspires real‐time sensing of some physical or chemical parameters of devices involved in bending behavior.

## 6 DESIGN OF ANGLE‐INDEPENDENT STRUCTURAL COLOR SENSING OF SPCSs

**FIGURE 16:**
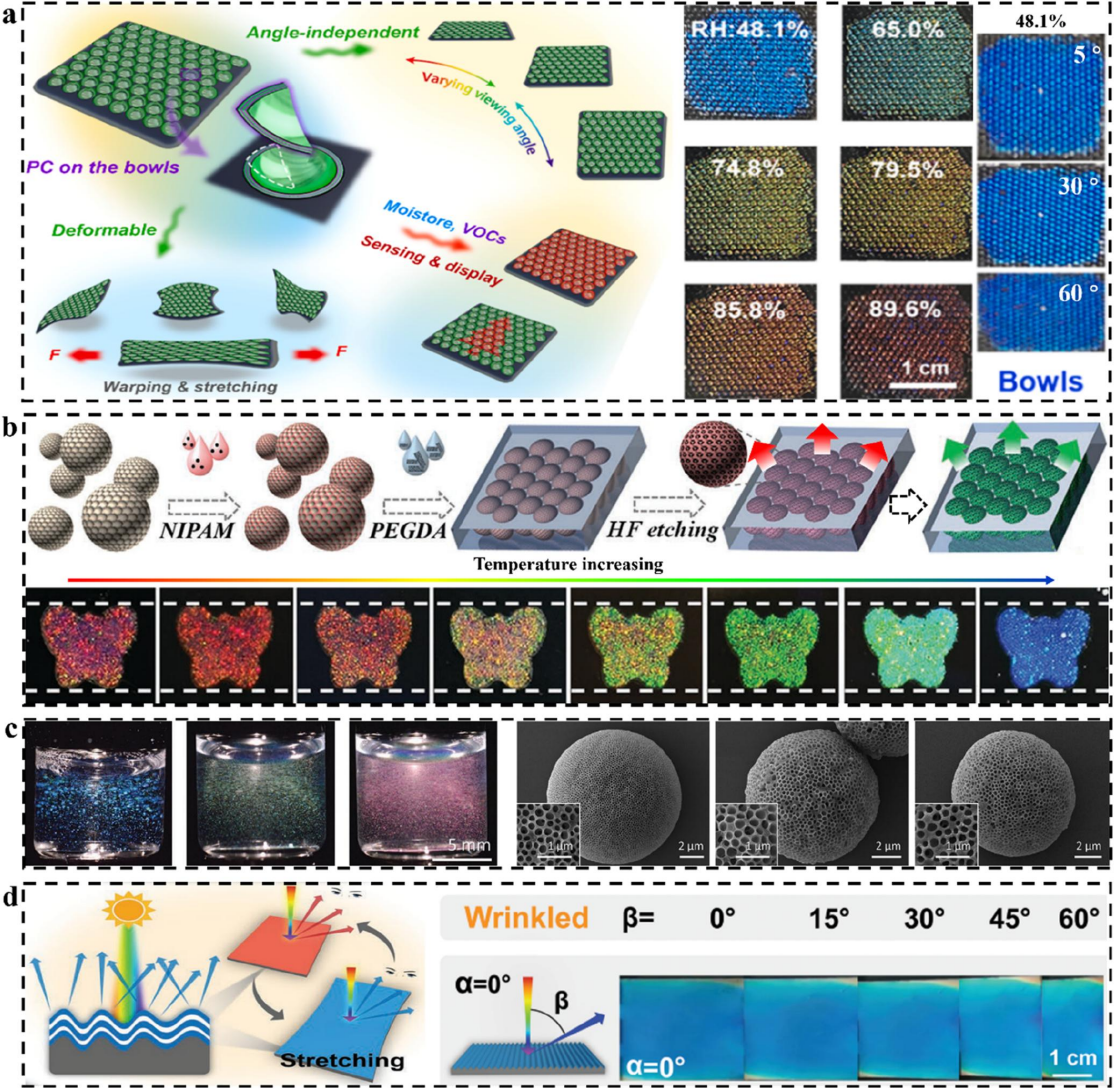
Swelling‐induced angle‐independent structural color. (a) Bowl‐array enabled angle‐independent 1D SPCS for environmental sensing,^[39]^ Copyright 2022, Elsevier; (b) The fabrication of angle‐independent structurally colored spherical hydrogels and its temperature sensing,^[278]^ Copyright 2021, Wiley‐VCH; (c) Spherical isotropic porous photonic pigment and corresponding porous structures,^[279]^ Copyright 2020, Wiley‐VCH; (d) Angle‐independent structural color obtained through surface wrinkling,^[40]^ Copyright 2022, Wiley‐VCH.

The structural color of PCs is also related to the geometry of the substrates.[^274^, ^275^] PCs constructed on the surface of spherical substrates display angle‐independent structural color.^[276]^ PCs fabricated on cylindrical surfaces show angle‐independent structural colors in the direction of the circular section and angle‐dependent structural colors in the direction of the column.[^171^, ^277^] PCs prepared on the surface of concave substrates can also display angle‐independent structural colors. These angle‐independent structural colors make use of the geometric structure of the substrate. Regardless of the orientation of the incident, the corresponding incident angle of the part that shows the structural color is always maintained at ∼0°. Based on the geometric structure of the substrate, the structural color produced by the SPCSs in response to external stimuli will remain uniform, avoiding the sensing error caused by angle change. For example, Ma *et al.* prepared 1D PCs on a bowl‐like array based on a developed PDMS transfer printing method, displaying pixel array‐like angle‐independent structural colors (Figure 16a).^[39]^ Such a sensor shows consistent bright and glossy color in a wide viewing angle even twisting and stretching. The prepared SPCS avoids angle interference in humidity sensing, which is more convenient for non‐professionals to use related sensors. Combining the swelling of the polymer in 1D PCs in response to environmental stimuli, a series of angle‐independent structural color sensors have been developed.

For spherical PCs, the composition could be a polymer‐opal complex.^[280]^ The corresponding PC unit can also be etched to form spherical inverse opal PCs.^[281]^ The associated coloration and color change mechanisms are similar to that of common PCs. By integrating PNIPAm and reduced graphene oxide nanoparticles into a spherical PC, Zhao *et al.* obtained near‐infrared photothermal responsive spherical structural color hydrogels (Figure 16b).^[278]^ The addition of carbon nanotubes and the modularization of spherical hydrogels gave the SPCS electrothermal responsiveness. The related structural color change mechanism is due to the temperature‐induced volume change of PNIPAm hydrogel, which leads to the change of lattice spacing of inverse opals. This idea inspires designing angle‐independent structural color sensors. Photonic spheres composed of suitably sized macropores also show similar angle‐independent structural colors. By using “controlled micellization” self‐assembly of the amphiphilic bottlebrush block copolymer, Song, Vignolini, and Parker *et al.* prepared a series of spherical photonic pigments that display angle‐independent structural colors (Figure 16c).^[279]^ We highly recommend that readers read the corresponding work for inspiration.[^282^, ^283^, ^284^, ^285^] Moreover, quasi‐amorphous arrays are also a class of structural color materials with angle‐independent properties. Readers may refer to some pieces of representative literature.[^286^, ^287^, ^288^, ^289^, ^290^]

Moreover, surface wrinkling can disrupt the original angle dependence by destroying the flat structure of the PC. Using the geometry of the periodic structures of wrinkles, angle‐independent structural colors could be obtained in some specific directions (Figure 16d).^[40]^ The angle‐independent property in the vertical wrinkle direction was caused by two reasons: (1) the wrinkle destroys the long‐range order of the 1D PC plane and scatters the reflected light of 1D PC; (2) the wrinkle introduces convex and convex arcs on the surface of 1D PC, and the arc reflector makes the structural color show good consistency when changing the viewing angle. The reflected light cannot be scattered in the direction parallel to wrinkles. These properties, combined with PC properties such as polymer swelling‐induced structural color change, could be used to design a series of SPCSs with angle‐independent structural colors.

## 7 OUTLOOK AND CHALLENGES

In this review, we systematically discussed the LSP‐based SPCSs from the perspectives of analysis method selection, structure color sensing and polymer swelling mechanisms, design and construction of PC nanostructures, etc. The maximum reflection wavelength of PC changes caused by changes in *n*_eff_, lattice spacing, or incident/detection angles during swelling can only be detected by the human eye in the visible range. Therefore, the swelling degree of polymer‐based SPCSs should not be too large. The LSPs will not swell violently under solvent stimulation. The crosslinking density and the physical or chemical interchain binding force are the key factors to regulate the swelling of the polymer. We describe the roles of water molecules during the swelling of polymers, including free water, bound water, interstitial water, and semi‐bound water. Polymer swelling is mainly controlled by the polymer‐water interaction force and the elastic shrinkage force. The interaction between water molecules and polymers could be preliminarily understood by 2D correlation analysis of infrared spectroscopy. The polymer moves the embedded PC structures during the swelling process, which changes the lattice spacing and produces variational structural colors. Polymer‐based SPCSs, especially opal composite films, can move uniformly only when there is interaction force, otherwise elastic potential energy can only be released in the form of micro‐cracks.[^52^, ^194^] The structural color of such PCs will display no obvious change. By analyzing the 2D correlation infrared spectroscopy of opal‐ion elastomers, the typical force between PC structures and the polymer is elucidated, which enlightens readers to analyze the color change mechanism of PCs from the molecular point of view. Then, we summarized the working principle and practical application of the corresponding SPCSs according to the dimensions of PCs, including 1D, 2D, and 3D PCs. Excellent SPCS often needs to be carefully designed and screened in terms of nanostructures and materials to best meet the needs of the sensing. With the development of nanotechnology, the nanostructure creation of PCs will be diversified in the future. In addition, the geometric characteristics of the SPCS substrates also provide a control strategy for structural color sensing. For example, the geometric reflection path modulation of spherical,^[291]^ bowl‐shaped,^[39]^ columnar surfaces,^[292]^ and wrinkles^[293]^ provides a method for developing angle‐independent structural colors. The angle‐independent structural color will compensate for the sensing error caused by angle deviation, improving the practicality of SPCSs. We believe that based on the diversity of nanostructures and the universality of materials, SPCSs are bound to become the leader of smart sensors in the future.

To this end, we also expect more specific stimuli‐swelling materials and micro‐nano structure‐modulated color‐changing mechanisms to be applied to the construction of SPCSs. A large number of stimuli‐responsive micro‐nano structures will play important roles in SPCSs, possibly including 2D nano‐to‐micro structures,[^37^, ^294^] surface wrinkles,[^295^, ^296^] photonic porous microparticles,[^279^, ^285^] quasiamorphous structures,[^289^, ^297^] diffraction gratings,[^298^, ^299^] bilayer photonic structures,[^26^, ^82^] photonic microcapsules,[^208^, ^275^] stratified porous structures,^[300]^ particle‐nested nanostructures,[^271^, ^301^] periodic wavy structures,^[302]^ woodpile PCs,^[303]^ micro‐sized bowl‐array,^[39]^ core‐shell particles,^[304]^ microbubbles,^[305]^ polyhedron particle‐arrays,^[306]^ and so on. Moreover, some novel sensing‐material systems, such as cholesteric liquid crystal[^307^, ^308^, ^309^] and blue‐phase liquid crystal,[^310^, ^311^, ^312^] also show extraordinary sensing properties. By limiting the horizontal diffusion of ink on the surface of such blue‐phase liquid crystal through hydrophobic treatment, the resolution of the printed pattern could be further improved.^[313]^ The structural color of the printed pattern is controlled by changing the lattice spacing through swelling. We predict that the innovation of high‐resolution structural color patterns will bring new ideas for the development of SPCSs in the future. Future SPCSs will inherit and extend the advantages of optical sensing and play important roles in many intersecting areas, such as visual degradation of polymers revealed by structural colors,^[314]^ multiple optical and shape encryption by the integration of structural/chemical colors and swelling polymers,^[315]^ structural color sensors regulated by swelling mechanisms in the ethanol industry,^[154]^ cell/drug activity sensing in biomedical engineering,^[88]^ etc.

The LSP allows only small volume changes and does not introduce new impurities into the systems. Sensors could be implanted and separated quickly, with little impact on the reaction system in many interdisciplinary areas. For example, during the chemical reaction process, the structural color sensor can accurately identify the micro‐reaction process and reveal the gas‐liquid mass transfer process on the material surface in real‐time.^[192]^ During the process of electrochemical reaction, the swelling and shrinkage of the skeleton caused by oxidation‐reduction reaction must lead to a change of structural color.[^166^, ^316^] The corresponding reaction kinetics and categories could be indicated accordingly. In the field of bioengineering, it is envisaged that cancer cells attached to the surface of SPCS secrete or metabolize active molecules (such as biothiols),^[317]^ and the related molecules break the disulphide bonds of the polymer hydrogels to reduce the degree of crosslinking,^[180]^ resulting in swelling of the polymer and color change of the PCs. There is no doubt that cell activity and real‐time metabolism could be monitored according to the structural color. By incorporating the swelling properties of polymers, future SPCSs could be directly used to construct porous microreactors with adjustable chemical and physical properties.[^192^, ^318^, ^319^] Of course, SPCS, as a medium to connect other disciplines and optical sensors, it is necessary to find out the corresponding color‐parameter transition method and controllable sensing mechanisms.

According to the working principle of SPCSs, the structural color sensor should be designed and constructed from at least four directions, including: (1) Whether the shape or arrangement distribution of micro‐nano structures could be changed with the swelling of the polymer? (2) How do the polymer molecular structures regulate the corresponding swelling process? (3) How to integrate PC structures into the target system more cleverly to make use of PC's porous structures and structural color? (4) How to improve the sensitivity of SPCSs and manipulate the angle‐dependent structural color? There are many challenges in developing SPCSs with controllable swelling performance, multimodal temporary state locking, good swelling removal capability, and adjustable angle dependence. In addition, several generalizations need to be considered, (1) How can LSPs be used to extend the structural color and porous structure of SPCSs to other research fields, such as porous catalytic carriers and targeted nanocrymers? (2) How to extend the light manipulation performance of PCs to human daily life? (3) How to reduce the commercialization cost of PCs? Because most artificial photonic structures are currently limited to the laboratory stage, it is necessary to think about how to be more satisfactory in terms of manufacturing cost, time consumption, sample reproducibility, and spatial resolution. We are optimistic that with the rapid development of advanced nanomanufacturing technologies, combined with the inspiration of nature,[^320^, ^321^, ^322^, ^323^, ^324^] these challenges could be solved in the future. We anticipate that this review will provide guidance for the design, fabrication, and application of SPCSs and stimulate new thinking and cutting‐edge innovation in multidisciplinary fields such as applied physics, materials science, biomedicine, chemical engineering, catalysis, and interface engineering.

## CONFLICT OF INTEREST STATEMENT

The authors declare no conflict of interest.

## Data Availability

The data that support the findings of this study are available from the corresponding author upon reasonable request.
